# Multiple downy mildew effectors target the stress‐related NAC transcription factor LsNAC069 in lettuce

**DOI:** 10.1111/tpj.14383

**Published:** 2019-07-04

**Authors:** Claudia‐Nicole Meisrimler, Alexandra J. E. Pelgrom, Bart Oud, Suzan Out, Guido Van den Ackerveken

**Affiliations:** ^1^ Plant–Microbe Interactions Department of Biology Utrecht University Padualaan 8 3584 CH Utrecht the Netherlands; ^2^ University of Canterbury Ilam Private Bag 4800 Christchurch 8041 New Zealand; ^3^ Enza Zaden Haling 1‐E Enkhuizen 1602 DB the Netherlands

**Keywords:** NAC transcription factors, oomycetes, drought stress, transcription factor relocalization, *Lactuca sativa*, *Bremia lactucae*, *Pseudomonas cichorii*

## Abstract

To cause disease in lettuce, the biotrophic oomycete *Bremia lactucae* secretes potential RxLR effector proteins. Here we report the discovery of an effector‐target hub consisting of four *B. lactucae* effectors and one lettuce protein target by a yeast‐two‐hybrid (Y2H) screening. Interaction of the lettuce tail‐anchored NAC transcription factor, LsNAC069, with *B. lactucae* effectors does not require the N‐terminal NAC domain but depends on the C‐terminal region including the transmembrane domain. Furthermore, in Y2H experiments, *B. lactucae* effectors interact with Arabidopsis and potato tail‐anchored NACs, suggesting that they are conserved effector targets. Transient expression of RxLR effector proteins BLR05 and BLR09 and their target LsNAC069 *in planta* revealed a predominant localization to the endoplasmic reticulum. *Phytophthora capsici* culture filtrate and polyethylene glycol treatment induced relocalization to the nucleus of a stabilized LsNAC069 protein, lacking the NAC‐domain (LsNAC069^Δ^ ^NAC^ ). Relocalization was significantly reduced in the presence of the Ser/Cys‐protease inhibitor TPCK indicating proteolytic cleavage of LsNAC069 allows for relocalization. Co‐expression of effectors with LsNAC069^Δ^ ^NAC^ reduced its nuclear accumulation. Surprisingly, *LsNAC069* silenced lettuce lines had decreased *LsNAC069* transcript levels but did not show significantly altered susceptibility to *B. lactucae*. In contrast, *LsNAC069* silencing increased resistance to *Pseudomonas cichorii* bacteria and reduced wilting effects under moderate drought stress, indicating a broad role of *LsNAC069* in abiotic and biotic stress responses.

## Significance Statement

To infect lettuce, the obligate biotrophic oomycete *Bremia lactucae* secretes a large arsenal of RXLR‐effector proteins. We show that four RXLR‐like effectors target the endoplasmic reticulum‐associated host LsNAC069 transcription factor at its membrane‐associated C‐terminal region and hinder immune stress‐induced relocalization to the nucleus. We reveal membrane‐associated NACs as conserved effector targets to reduce immune activation. Furthermore, *LsNAC069* is not only involved in plant immunity but also in drought stress adaptation.

## Introduction

Plant pathogenic downy mildews and *Phytophthora* spp. form a major threat to numerous economically important crops in agriculture. These filamentous oomycetes penetrate a variety of plant tissues and spread intercellularly through hyphal growth. Specialized feeding structures, the haustoria, are formed from hyphae, invade host cells, but remain separated from the plant cell cytoplasm by the plant‐derived extrahaustorial membrane (EHM). Haustoria form the main secretion site of effector molecules that act extracellularly (apoplastic effectors) or inside plant cells (host‐translocated effectors; Whisson *et al*., 2007, 2016). In the co‐evolutionary arms race between plants and pathogens, effector proteins are deployed to suppress plant immune responses. These can be triggered by detection of pathogen‐associated molecular patterns (PAMPs) resulting in pattern‐triggered immunity (PTI), or effectors leading to effector‐triggered immunity. Host‐adapted pathogens have evolved effectors to counter both layers of the plant immune system and induce a state of susceptibility (Jones and Dangl, 2006).

RxLR effectors, the main class of host‐translocated effectors in downy mildews and *Phytophthora* spp., are characterized by an N‐terminal signal peptide and a conserved RxLR (Arg – x – Leu – Arg) motif followed by a sequence diverse C‐terminal effector domain. Predicted RxLR effector repertoires range from dozens to several hundreds of proteins per species (Jiang *et al*., 2008; Haas *et al*., 2009; Fabro *et al*., 2011; Stassen *et al*., 2012). Yet, the molecular mechanisms by which most RxLR effectors affect plant immunity remains unknown.

The *Phytophthora infestans* RxLR effector Pi03192 was reported to interact with potato (*Solanum tuberosum*) transcription factors (TFs) StNTP1 and StNTP2 at the endoplasmic reticulum (ER; McLellan *et al*., 2013). The StNTPs belong to the NAC [no apical meristem (NAM), *Arabidopsis thaliana* transcription activation factor (ATAF1/2) and cup‐shaped cotyledon (CUC2)] family of TFs. StNTP1 and StNTP2 are released from the ER upon *P. infestans* culture filtrate (CF) treatment and relocalize to the nucleus. Interestingly, relocalization of StNTP1 and StNTP2 was inhibited in the presence of effector Pi03192 and silencing of StNTP orthologs in *Nicotiana benthamiana* increased susceptibility to *P. infestans* infection, suggesting that these NAC TFs play a role in plant immunity and disease resistance (McLellan *et al*., 2013).

StNTP1/2 belong to a NAC family subgroup that contains an N‐terminal NAC domain and a C‐terminal transmembrane domain (TMD). In Arabidopsis, 14 of the 117 putative NAC genes contain a functional TMD (Kim *et al*., 2007a; Klein *et al*., 2012; Yang *et al*., 2014; Liang *et al*., 2015; Zhao *et al*., 2016). Most of these tail‐anchored TFs localize to the ER (Klein *et al*., 2012; Block *et al*., 2014; Liang *et al*., 2015), and a minority to the plasma membrane (Liang *et al*., 2015; Zhao *et al*., 2016).

Tail‐anchored TFs are a subgroup of membrane‐associated TFs that enable plant cells to respond rapidly to developmental and environmental cues by switching from a membrane‐tethered dormant state to a transcriptionally active form upon proteolytic cleavage. Proteolysis can be initiated by a variety of triggers, including cold exposure, high salinity, mitochondrial stress and ER stress (Kim *et al*., 2007b, 2008; Seo *et al*., 2010; De Clercq *et al*., 2013; Ng *et al*., 2013). After cleavage, the TF relocalizes to the nucleus to regulate gene expression.

In this study, we focus on one lettuce effector target of the downy mildew *Bremia lactucae*. This obligate biotrophic pathogen poses a major threat to lettuce cultivation worldwide. Analysis of the *B. lactucae* transcriptome resulted in identification and cloning of 49 RXLR‐like effectors and one Crinkler (Stassen *et al*., 2012, 2013; Giesbers *et al*., 2017). Multiple *B. lactucae* RxLR effectors are recognized in specific lettuce accessions (Stassen *et al*., 2013; Giesbers *et al*., 2017; Pelgrom *et al*., 2019), but no effector targets have been described yet. We identified the tail‐anchored NAC, LsNAC069, as target of four *B. lactucae* RXLR‐like effectors. Target and effectors localize to the secretory pathway *in planta* and LsNAC069‐effector‐interaction required the C‐terminal region including the TMD, but not the NAC domain. Relocalization of LsNAC069 was induced by treatment with *Phytophthora capsici* CF and polyethylene glycol (PEG), but was inhibited upon co‐expression of *B. lactucae* effectors and the protease inhibitor TPCK. We conclude that LsNAC069 relocalization from the ER to the nucleus is triggered upon immunity activation as well as during osmotic stress, and *B. lactucae* uses effector proteins to inhibit this relocalization process, ultimately hindering activation of LsNAC069 downstream genes.

## Results

### A lettuce NAC transcription factor interacts with four *Bremia lactucae* effectors

**Figure 1:**
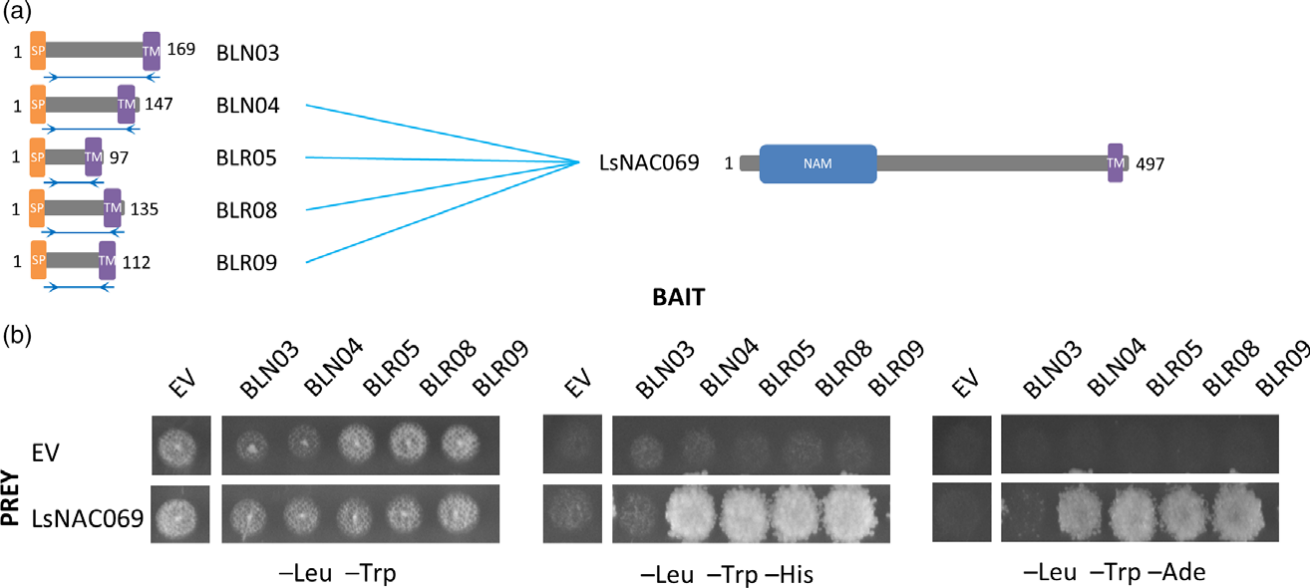
Four *Bremia lactucae* RxLR effectors converge on LsNAC069. (a) Graphical representation of five *B. lactucae* effectors and the target LsNAC069. Predicted domains are shown in different colors. Double blue arrows indicate the region used as bait in yeast‐two‐hybrid (Y2H). (b) Y2H of effectors BLN03, BLN04, BLR05, BLR08 and BLR09 and full‐length LsNAC069, which activates both reporter genes. From left to right: permissive (−Leu −Trp), moderate (−Leu −Trp −His) and strongly (−Leu −Trp −Ade) selective medium. EV, empty pDEST32 (bait) or pDEST22 (prey) vector; SP, signal peptide; TM, transmembrane domain.

Yeast‐two‐hybrid (Y2H) screens were performed using a lettuce cDNA prey library with the *B. lactucae* effectors BLN03^22‐169^, BLN04^24‐147^, BLR05^22‐97^, BLR08^30‐135^ and BLR09^23‐112^, that each contain a single C‐terminal TMD (Figure 1a) as bait. Four of these, BLN04, BLR05, BLR08 and BLR09, interacted with the same lettuce target, a NAC TF (Lsat_1_v5_gn_6_99960.1, gene identifier used in http://lgr.genomecenter.ucdavis.edu/; hereafter LsNAC069). Five additional interactors were found for BLR05, three for BLR08 and six for BLR09 (see Table S1 for all identified interactions). In multiple prey clones encoding LsNAC069 coding sequences were present from the start codon, whereas other clones only contained the 3′ half encoding the C‐terminal part of the protein (Figure S1). To confirm if LsNAC069 interacts as full‐length protein with the different effectors, prey plasmids containing the complete coding sequence were co‐transformed with the effector bait plasmids in yeast. Full‐length LsNAC069 consistently interacted with effectors BLN04, BLR05, BLR08 and BLR09, but not BLN03.

### Effectors and LsNAC069 localize to the endoplasmic reticulum *in planta*

**Figure 2:**
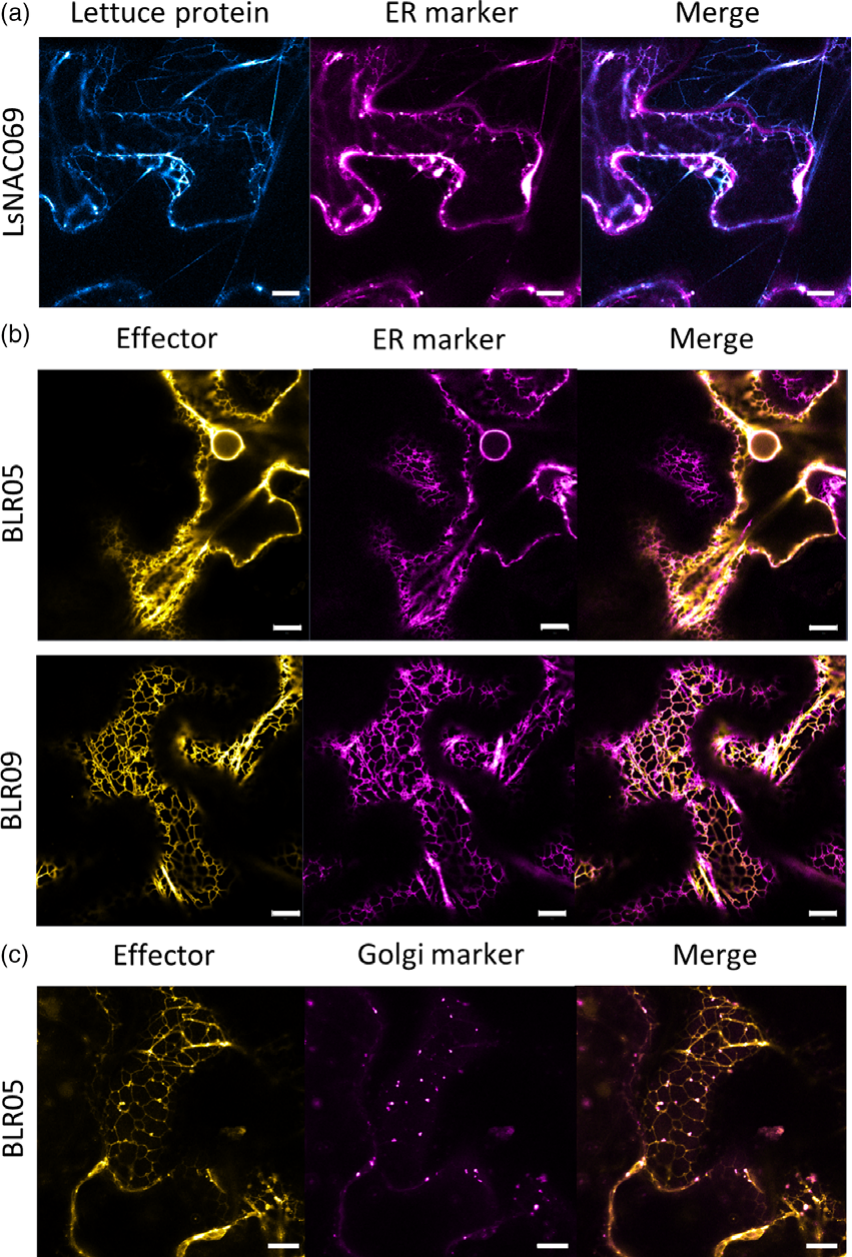
LsNAC069 and effectors BLR05 and BLR09 localize to the endoplasmic reticulum (ER). Fluorescent fusions of LsNAC069 and effector proteins were transiently expressed in *Nicotiana benthamiana*. (a) CFP‐LsNAC069 co‐localizes with RFP‐KDEL ER marker. (b) YFP‐BLR05 and YFP‐BLR09 co‐localize with CFP‐ER marker. (c) YFP‐BLR05 co‐localizes with CFP‐Golgi marker. Scale bars indicate 10 μm.

During *B. lactucae* infection of lettuce the effectors and target were found to be expressed, suggesting that the encoded proteins can be found in the same tissues and cells (Figure S2). To localize the proteins *in planta*, N‐ and C‐terminal fluorophore (CFP, YFP or RFP)‐tagged effectors and LsNAC069 (Karimi *et al*., 2005) were transiently expressed in *N. benthamiana* and analyzed by confocal laser‐scanning microscopy (CSLM). Whereas most fusion proteins could be well visualized, the fluorophore fusions of BLN03 and BLN04, both the N‐ and C‐terminal ones, were undetectable. CFP‐LsNAC069, YFP‐BLR05 and YFP‐BLR09 labeled an intracellular reticulate compartment, which was confirmed to be the ER by subsequent co‐expression with the luminal ER marker HDEL fusion to CFP or RFP (Nelson *et al*., 2007; Figure 2a,b). Punctate structures closely associated with the ER in YFP‐BLR05 expressing cells were identified as Golgi bodies (Figure 2c; Nelson *et al*., 2007). Strikingly, YFP‐ and RFP‐BLR08 labeled unidentified ring‐like structures of varying sizes (up to 10 μm in diameter) suggesting that BLR08 induced cell stress (Figure S3).

To determine if the effectors BLR05, BLR09 and LsNAC069 proteins show the same localization in the original host system, the fluorophore fusions were transiently expressed in lettuce. Also here, BLR05, BLR09 and the NAC protein localized to the ER (Figure S4), confirming the observations in *N. benthamiana*. Overall, our CLSM data support the *in planta* localization of BLR05, BLR09 and their target LsNAC069 to the ER.

### Phylogenetic analysis of lettuce NAC transcription factors and plant tail‐anchored NACs

NAC TFs occur in vast gene families in land plants with 117 genes in Arabidopsis, 74 in grape, 110 in potato, 151 in rice, and 152 in soybean and tobacco (Rushton *et al*., 2008; Nuruzzaman *et al*., 2010; Le *et al*., 2011; Singh *et al*., 2013; Wang *et al*., 2013; Pereira‐Santana *et al*., 2015). The recently published *Lactuca sativa* cv. Salinas genome (Reyes‐Chin‐Wo *et al*., 2017) was used to characterize the lettuce NAC gene family and position LsNAC069 herein. We identified 94 NAC family members in lettuce (Table S2), and the proteins could be divided over nine subfamilies (Figure S5).

**Figure 3:**
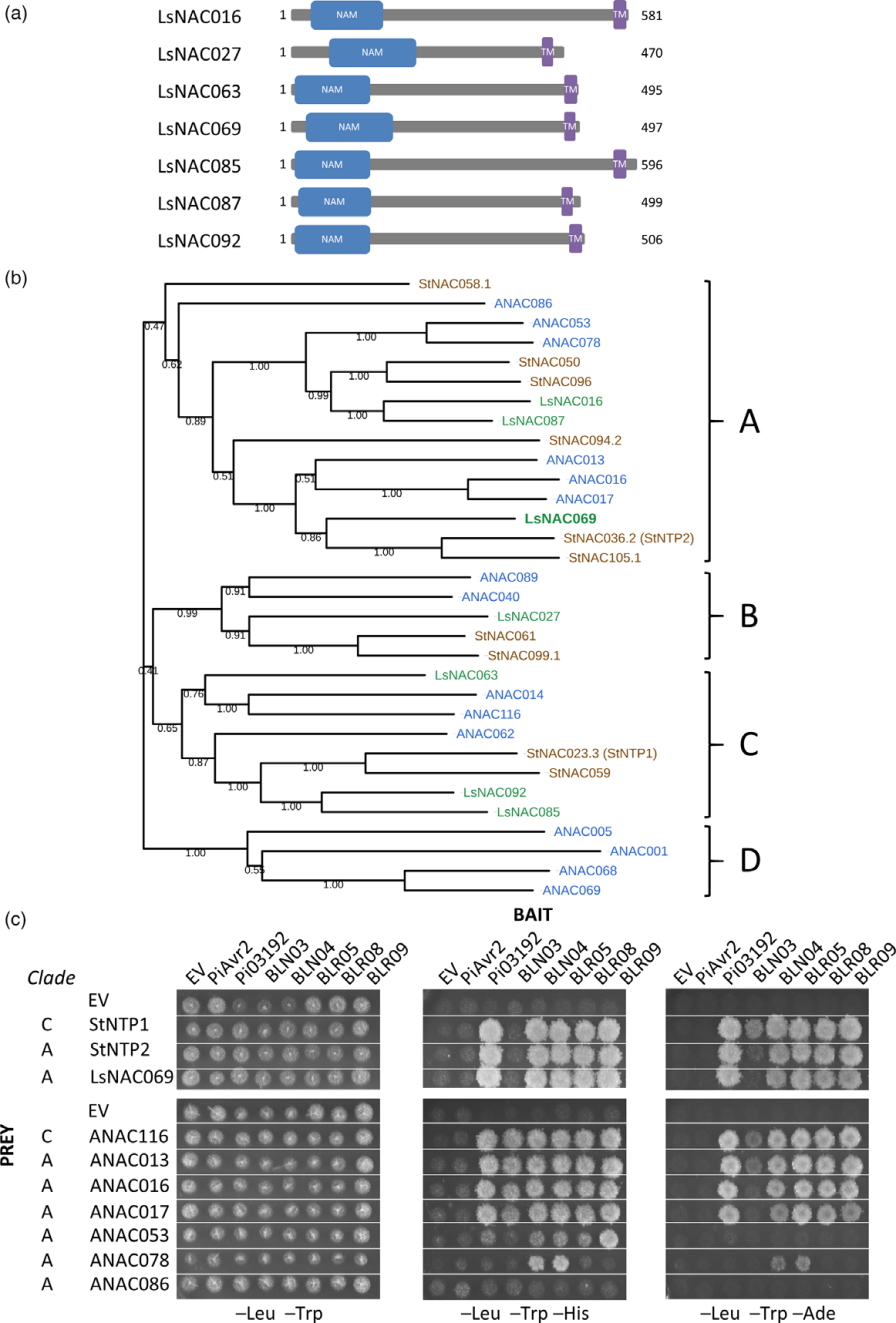
Tail‐anchored NACs are conserved effector targets. (a) Graphical representation of lettuce NACs with putative single C‐terminal transmembrane domain (TMD; purple) and N‐terminal NAM domain (blue). (b) Phylogeny of tail‐anchored NACs from lettuce (green), potato (brown) and Arabidopsis (blue). Clades A–D are indicated on the right. (c) Interaction of *Phytophthora infestans* effectors PiAvr2, Pi03192, *Bremia lactucae* effectors BLN03, BLN04, BLR05, BLR08, BLR09 and potato, lettuce and Arabidopsis tail‐anchored NACs. From left to right: permissive, moderate and strongly selective plate. EV, empty pDEST32 (bait) or pDEST22 (prey) vector.

LsNAC069 meets the requirements for a tail‐anchored protein, as it does not contain an N‐terminal signal peptide, but is targeted and anchored to the membrane via a single TMD in the last approximately 40 C‐terminal amino acids (Pedrazzini, 2009; Borgese and Fasana, 2011). All lettuce NAC proteins were analyzed for the presence of single C‐terminal TMDs, resulting in a total of seven putative tail‐anchored NAC proteins (Figures 3a and S5), including LsNAC069, and all of which clustered together within subfamily 9.

To explore the phylogenetic relationship of LsNAC069 to tail‐anchored NAC TFs of other plant species, a phylogenetic tree was constructed that included 7 lettuce, 14 Arabidopsis and 10 potato tail‐anchored NAC TFs (Figure 3b). The potato tail‐anchored NACs included StNTP1 and StNTP2, previously identified as targets of the *P. infestans* effector Pi03192 (McLellan *et al*., 2013). Tail‐anchored NACs from lettuce, Arabidopsis and potato cluster in four distinct clades. LsNAC069 was found in clade A grouping with orthologs StNTP2, StNAC105, ANAC016 and ANAC017. StNTP1 is in clade C with StNAC059 and three Arabidopsis and lettuce tail‐anchored NACs. Clade D formed an exception from the other three clades as it only contains Arabidopsis paralogous proteins, suggesting a lineage‐specific expansion.

### Plant tail‐anchored NACs are conserved interactors of pathogen effectors

The close phylogenetic relationship of tail‐anchored NAC proteins raised the possibility that they share a conserved effector‐interaction site. To determine if the potato StNTP1 and StNTP2 can interact with *B. lactucae* effectors and LsNAC069 can interact with the *P. infestans* effector Pi03192, a targeted Y2H screen was performed. LsNAC069, StNTP1 and StNTP2 all interacted with Pi03192, but not with the unrelated *P. infestans* effector PiAvr2 (Figure 3c). Also, StNTP1 and StNTP2 interacted strongly with BLN04, BLR05, BLR08 and BLR09 (Figure 3c). Thus, LsNAC069, StNTP1 and StNTP2 appear to interact with the same set of, sequence diverse, effectors.

To determine if the effector‐interaction is also observed with other tail‐anchored NACs, we tested six Arabidopsis proteins in the Y2H system (Figures 3c and S6). The soluble ANAC086 (not containing a TMD) that groups with the tested Arabidopsis tail‐anchored NACs (Shen *et al*., 2009) was included in the Y2H, but did not interact with any of the effectors. ANAC013, ANAC016 and ANAC017, clustering together with LsNAC069 and StNTP2 in clade A (Figure 3b), interacted strongly with effectors Pi03192, BLN04, BLR05, BLR08 and BLR09, similarly to LsNAC069, but also interacted weakly with BLN03. ANAC116 (Nakano *et al*., 2006) that groups with StNTP1 in clade C also interacted with all five *B. lactucae* effectors in the Y2H assay. In contrast, ANAC053 and ANAC078 of clade A (Figure 3b) with a higher sequence similarity to LsNAC069 and StNTP2 than StNTP1 were interacting poorly. This result implies that the specificity of the effector‐NAC‐interaction cannot be reduced to overall sequence similarity. Summarizing, the protein interaction data show that plant tail‐anchored NACs are conserved interactors of oomycete effectors.

### The LsNAC069 C‐terminal domain is required for effector‐interaction

**Figure 4:**
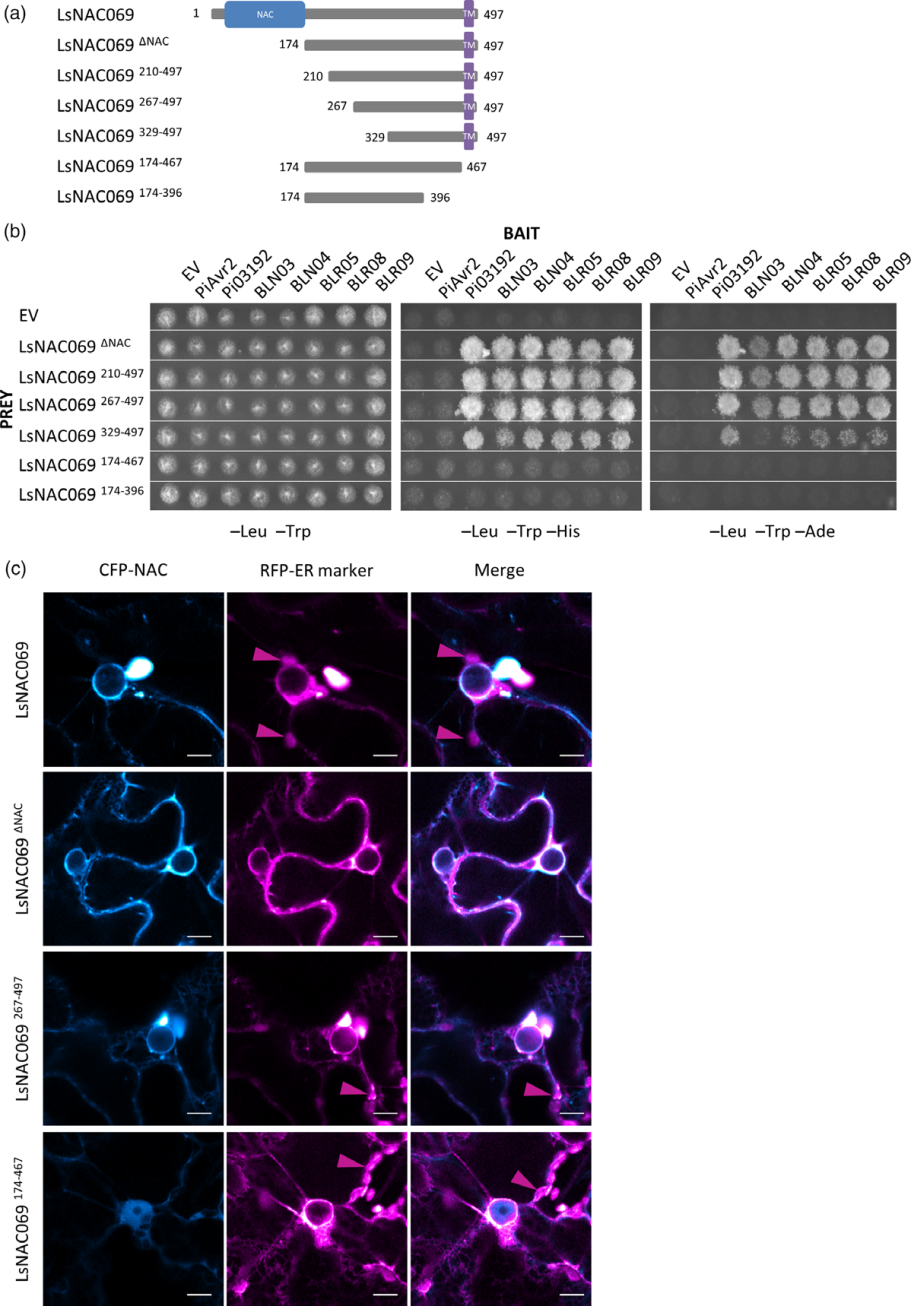
Effector‐target interaction requires the transmembrane domain (TMD) but not the NAC domain. (a) Graphical representation of the truncated LsNAC069 constructs. NAC domain is shown in blue and the TM domain in purple. (b) Interaction between *Phytophthora infestans* effectors PiAvr2, Pi03192, *Bremia lactucae* effectors BLN03, BLN04, BLR05, BLR08, BLR09 and truncated LsNAC069 proteins. Removal of the NAC domain and parts of the variable domain are well tolerated, but truncation of the TMD abolishes interaction. From left to right: permissive (−Leu −Trp), moderate (−Leu −Trp −His) and strongly (−Leu −Trp −Ade) selective medium. EV, empty pDEST32 (bait) or pDEST22 (prey) vector. (c) CFP‐tagged LsNAC069, LsNAC069^Δ^ ^NAC^ and LsNAC069^267‐497^ co‐localize with the RFP‐KDEL ER marker, whereas LsNAC069^174‐467^ localizes solely nuclear‐cytosolic. Full LsNAC069 induces cell stress and deformation of the nucleus (Figure S8). All samples were treated with MG132. Purple arrows indicate autofluorescence of chloroplasts. Scale bars indicate 10 μm.

To map the part of LsNAC069 required for interaction with the effectors, multiple N‐ and C‐terminal truncated constructs were generated (Figure 4a) and tested for interaction in the Y2H system. Removal of the NAC domain in LsNAC069^174‐497^ (hereafter LsNAC069^ΔNAC^) and LsNAC069^267‐497^ did not abolish interaction with the effectors (Figure 4b), indicating that the conserved NAC domain is not required for effector binding. This is corroborated by the fact that 14 of the 31 original Y2H identified prey clones with LsNAC069 fragments lacked the NAC domain (Figure S1). Even LsNAC069^329‐497^, containing only the C‐terminal portion of the protein, was still able to interact, although weaker. Compared with full‐length LsNAC069, the N‐terminally truncated constructs also interacted moderately with BLN03 as the corresponding yeast transformant still grew weakly on –Leu –Trp –Ade medium (Figure 4b). In contrast, removal of the C‐terminal TMD in LsNAC069^174‐467^ and LsNAC069^174‐396^ completely abolished interaction with all tested baits (Figure 4b), indicating that the C‐terminal region is essential for effector binding.

As truncated proteins may display altered stability and/or subcellular localization *in planta*, N‐terminal CFP fusions of LsNAC069^ΔNAC^, LsNAC069^267‐497^ and LsNAC069^174‐467^ were transiently expressed in *N. benthamiana*. CFP‐LsNAC069^ΔNAC^ and CFP‐LsNAC069^267‐497^ both labeled the ER (Figure 4c) like CFP‐LsNAC069. CFP‐LsNAC069^ΔNAC^ did not induce cell stress‐based formation of ER bodies and vesicles observed during expression of CFP‐LsNAC069 and CFP‐LsNAC069^267‐497^ (Figure 4c). Furthermore, all truncated forms of LsNAC069 showed an increased stability compared to the full LsNAC069, as shown by CLSM and by protein immune precipitation combined with immunoblotting (Figures S7 and S8). LsNAC069^174‐467^, lacking the TMD, localized to the cytoplasm and nucleus with no visible ER association (Figures 4c and S8). The fluorescence signal and protein stability of LsNAC069 was increased by addition of the proteasome inhibitor MG132, whereas the truncated proteins remained stable independent of MG132 (Figures S7 and S8). Further experiments were therefore carried out with LsNAC069^ΔNAC^ and LsNAC069^174‐467^ fluorescent protein fusions.

### LsNAC069^ΔNAC^ co‐localizes with *Bremia lactucae* effectors *in planta*

**Figure 5:**
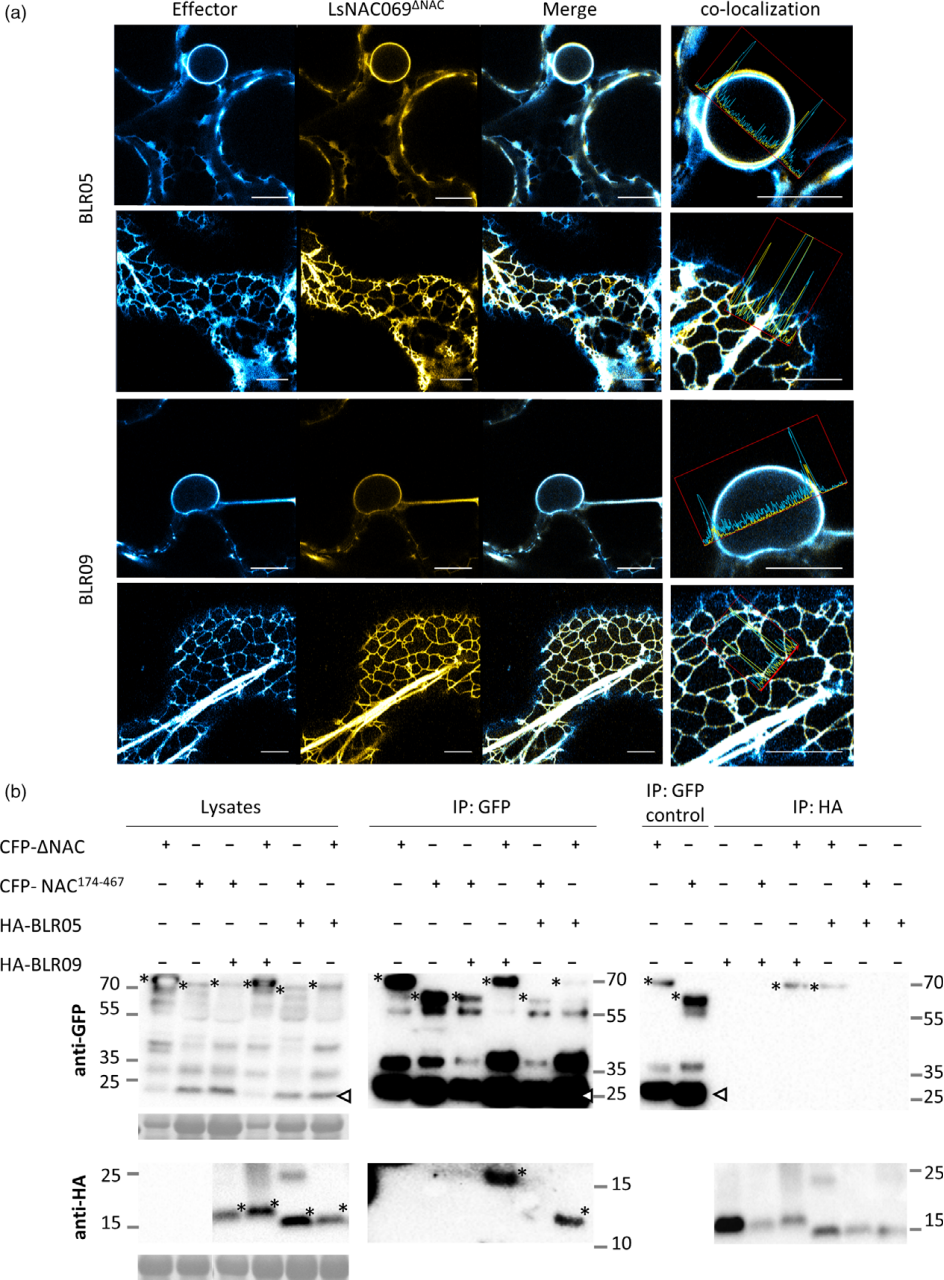
Co‐expression and co‐immunoprecipitation (co‐IP) of LsNAC069^Δ^ ^NAC^ with *Bremia lactucae* effectors in *Nicotiana benthamiana*. (a) CFP‐LsNAC069^Δ^ ^NAC^ co‐localizes with YFP‐BLR05 and RFP‐BLR09 at the endoplasmic reticulum (ER) membrane. Scale bars indicate 10 μm. (b) Co‐IP confirmed interaction of LsNAC069^Δ^ ^NAC^ with BLR05 and BLR09. Representative images of co‐IPs of LsNAC069^Δ^ ^NAC^ with HA‐BLR05 and HA‐BLR09 are shown. CFP‐NAC ^174‐467^ was used as the negative control. Co‐IP was performed in both directions, with anti‐GFP and anti‐HA beads. The asterisk indicates the monomeric sizes of proteins and the arrow indicates the monomeric size of free CFP.

The more stable CFP‐LsNAC069^ΔNAC^ was co‐expressed with *B. lactucae* effectors BLR05 and BLR09 in *N. benthamiana*. CFP‐LsNAC069^ΔNAC^ clearly co‐localized with YFP‐BLR05 and YFP‐BLR09 at the ER (Figures 5a, S9 and S10). We proceeded with co‐immunoprecipitation (co‐IP) of co‐expressed CFP‐LsNAC069^ΔNAC^ with HA‐tagged effectors, which confirmed interaction for LsNAC069^ΔNAC^ with HA‐BLR05 and HA‐BLR09 (Figure 5b). In contrast, the HA‐BLR08‐LsNAC069^ΔNAC^‐interaction could not be confirmed. Therefore, we decided to focus further experiments on the confirmed effector proteins BLR05 and BLR09. It can be summarized that BLR05 and BLR09 co‐localize and interact with LsNAC069^ΔNAC^ in plants, supporting the hypothesis that these two effectors target LsNAC069 during *B. lactucae* infection.

### *Bremia lactucae* effectors inhibit relocalization of LsNAC069 to the nucleus

**Figure 6:**
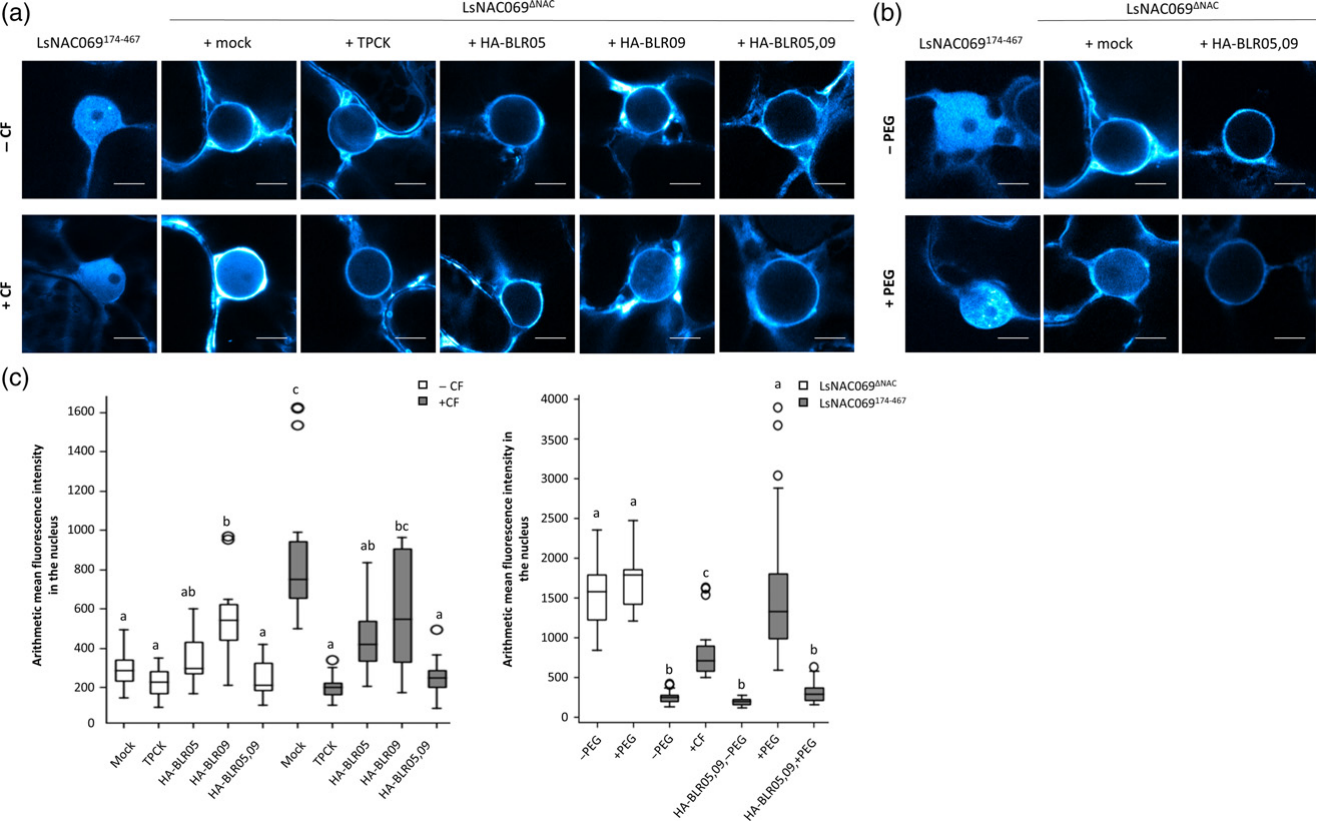
Nuclear accumulation of LsNAC069^Δ^ ^NAC^ is induced by *Phytophthora capsici* culture filtrate (CF) and polyethylene glycol (PEG), but is inhibited in the presence of TCPK or combined *Bremia lactucae* effectors. (a, b) Localization of CFP‐LsNAC069^Δ^ ^NAC^ and CFP‐LsNAC069^174‐467^ in *Nicotiana benthamiana* leaf sections. All samples were treated with proteasome inhibitor MG132. Confocal settings were identical between samples. The scale bar indicates 10 μm. (a) Leaf sections treated with *P. capsici* CF show a clear relocalization of LsNAC069^Δ^ ^NAC^ into the nucleus in comparison to the control (‐CF). Protease inhibitor TPCK decreases relocalization to the nucleus. A similar effect can be observed for co‐expression of HA‐BLR05 and HA‐BLR09. CFP‐LsNAC069^174‐467^, control without transmembrane domain (TMD), was unaffected by all treatments. (b) PEG (5% v/v) treatment induces relocalization of CFP‐LsNAC069^Δ^ ^NAC^ . Co‐expression of LsNAC069^Δ^ ^NAC^ with HA‐BLR05 and HA‐BLR09 inhibits PEG‐induced relocalization significantly. (c,d) Quantification of arithmetic mean fluorescence in the nucleus. Bars represent the mean SD from *n* ≥ 13 images per treatment. Statistical differences were assessed using one‐way anova with *post hoc* Tukey testing.

Membrane‐associated TFs, like the tail‐anchored NACs, quickly respond to stress signals by cleavage of the TMD, allowing relocalization to the nucleus. To induce stress responses, *N. benthamiana* can be treated with CF of germinating *Phytophthora* zoospores to increase expression of PTI genes as PAMPs in the CF activate plant immunity (McLellan *et al*., 2013; Yang *et al*., 2016). To assess if CF can trigger relocalization of LsNAC069, *N. benthamiana* leaf sections were incubated with CF of *P. capsici* in the presence of the proteasome inhibitor MG132. CF triggered the relocalization of CFP‐LsNAC069^ΔNAC^ to the nucleus, whereas MG132 treatment alone did not (Figure 6a). Also, the medium control did not induce relocalization (Figure S8). The localization of LsNAC069^174‐467^, without the TMD, was not affected by CF treatment as localization remained cytosolic and nuclear.

Previously, it was shown that ANAC017 cleavage was inhibited upon treatment with TPCK (Ng *et al*., 2013), a Ser/Cys protease inhibitor (Bond and Butler, 1987). When tested on CFP‐LsNAC069^ΔNAC^, TPCK affected the relocalization, retaining CFP‐LsNAC069^ΔNAC^ at the ER membrane even upon treatment with CF (Figure 6a). Proteolytic cleavage of CFP‐LsNAC069^ΔNAC^ is thus effectively inhibited by TPCK, suggesting that protease activity is required for relocalization.

As the *B. lactucae* effectors BLR05 and BLR09 interacted with the LsNAC069 C‐terminus including the TMD, one can envision that they prevent proteolytic cleavage of LsNAC069. Single co‐expression of BLR05 with CFP‐LsNAC069^ΔNAC^ reduced CF‐induced relocalization significantly, even though not as strong as co‐expression of combined effectors BLR05 and BLR09 (Figures 6a,c and S11). Western blots confirmed the presence of all tagged proteins in the experiments, indicating the comparability of the relocalization and the effect of the effector proteins (Figure S12). Summarizing, results show that activation of plant immunity induced relocalization of LsNAC069^ΔNAC^. The TPCK inhibitory effect suggests that cleavage is accomplished by a Ser or Cys protease, which can no longer exert its activity on LsNAC069 in the presence of multiple *B. lactucae* effectors.

### Silencing of *LsNAC069* increases resistance to bacteria but not downy mildew

**Figure 7:**
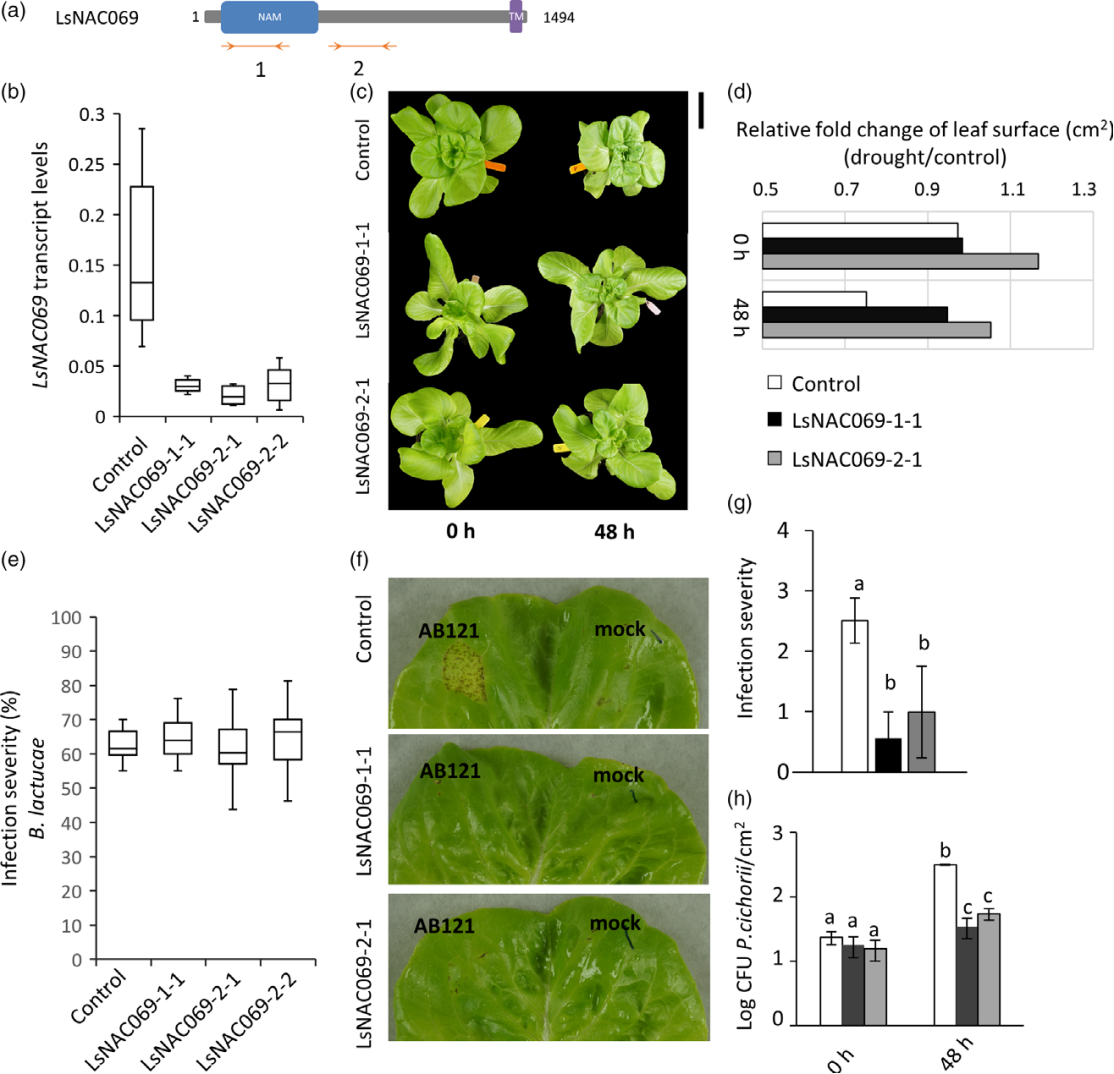
Silencing of *LsNAC069* does not result in significantly altered susceptibility to *Bremia lactucae*, but increases resistance to *Pseudomonas cichorii* and reduces wilting effects during drought stress. (a) Graphical representation of the two hpRNA target sites (double orange arrows) in LsNAC069. Transcript levels of (b) LsNAC069 in untransformed Wendell plants (control) and T2 lines harboring hpRNA construct 1 and 2 (two independent lines shown). Transcript levels are relative to lettuce ACTIN (Lsat_1_v5_gn_8_116260.1). Each boxplot contains the values of four–six plants. (c,d) Drought stress effect on RNAi lines (T3) at 0 and 48 h, with *n* ≥ 6 biological replicates. Scale bar indicates 10 cm. (c) Shown is the representative phenotype and (d) the relative fold change of the leaf surface area in cm^2^ of drought stress‐treated lines normalized relative to control‐treated lines. Due to wilting effects during drought stress, the measured surface of the control plants decreases more than the surface of the RNAi lines. This effect is caused by the loss of turgor and can be used as an indicator for a wilting phenotype. (e) Susceptibility to race Bl:24 infection at 11 dpi. Infection severity was scored as the percentage of the leaf disc surface area covered in sporangiophores. Each boxplot contains the values of 10–20 plants. (f–h) *Phytophthora cichorii* (AB121) assay in 5‐week‐old plants (T3). (f) Leaves 48 h after *P. cichorii* infiltration. (g) Severity of *P. cichorii* infection 48 h after infiltration at a scale of 0–4, with 0 = no visible effect to 4 = fully rotten. (h) Colony‐forming units on plates of dilution series of infected leaves at 0 and 48 h after infiltration (six independent leaves).

The role of *LsNAC069* in disease susceptibility of lettuce was further investigated in three independent *LsNAC069*‐silenced transgenic *L. sativa* cv. Wendell lines (Figure 7). Gene silencing efficiency of the hpRNA constructs, assessed by quantitative polymerase chain reaction (qPCR), was approximately 80% (Figure 7a). Transcript levels of LsNAC091, the most closely related gene within the hpRNA‐targeted region, was not affected, demonstrating that the hpRNA constructs are specific (Figure S13).

Susceptibility of the silenced lines to downy mildew infection was determined at 11 days post‐infection. Untransformed Wendell plants were highly susceptible to *B. lactucae*, with infection severity ranging between 50% and 80%. LsNAC069‐silenced plants displayed slightly enhanced infection in subsets of plants, but did not increase significantly over the whole experiment (Figure 7e). Hence, we conclude that silencing of *LsNAC069* alone does not significantly alter susceptibility to *B. lactucae*. To further explore the potential role of *LsNAC069* in plant immunity, 5‐week‐old plants were assessed for susceptibility to the bacterium *Pseudomonas cichorii*. This Gram‐negative bacterium infects a wide range of hosts and has an important economic impact on greenhouse‐grown lettuce. It is one of the bacteria known to induce lettuce midrib rot (Pauwelyn *et al*., 2011). The first appearance of symptoms involves water‐soaked lesions that develop in the infected leaf area and progressively turn black or brown. Two days after inoculation, disease symptoms, as well as bacterial growth, were significantly reduced in RNAi lines compared with the control Wendell plants (Figure 7f–h), suggesting that in wild‐type plants LsNAC069 negatively affects immunity to *P. cichorii*.

### Does LsNAC069 also play a role in abiotic stress?

During seed propagation of the LsNAC069 silenced lines, we observed an increased resistance to moderate drought stress. Recently, NACs were shown to be involved in drought stress and pathogen resistance (Wang *et al*., 2017), therefore we tested the potential effect of drought stress on LsNAC069 nuclear relocalization. For this experiment, we used PEG treatment of the plant cells in a similar manner as the CF treatment (Figure 6b). The osmolyte PEG is regularly used to induce drought stress in hydroponic systems (Hellal *et al*., 2018). PEG was found to be a stronger activator of the CFP‐LsNAC069^ΔNAC^ relocalization mechanism than *Phytophthora* CF (Figure 6d). In additional experiments, we co‐expressed the effectors BLR05 and BLR09 with CFP‐LsNAC069^ΔNAC^ and compared PEG‐treated and untreated samples for NAC‐relocalization. BLR05 and BLR09 each significantly inhibited the PEG‐induced relocalization of CFP‐LsNAC069^ΔNAC^ to the nucleus. The observed inhibitory effect was further increased upon combined expression of both effectors (Figures 6b,d and S14).

Because PEG induced LsNAC069 relocalization, a drought stress experiment was performed with 4‐week‐old lettuce plants. The experiment was conducted with *LsNAC069*‐silenced transgenic *L. sativa* cv. Wendell lines (Figure 7a) and untransformed Wendell plants as control. No phenotypic differences were observed at 0 and 24 h of ceased watering. At 48 h, Wendell plants showed increased wilting in comparison with two RNAi lines (Figure 7c,d). Interestingly, after 72 h phenotypic differences were less noticeable (Figure S15), suggesting a role of LsNAC069 in short moderate drought stress but not in continuous drought stress.

## Discussion

### *Bremia lactucae* effectors interact with the C‐terminal region of the endoplasmic reticulum‐associated LsNAC069

BLR05, BLR08 and BLR09 are canonical RXLR effectors, whereas BLN03 and BLN04 lack the RXLR motif but do contain an EER motif (Figure 1). Although the effectors do not display overall sequence homology, strikingly, they all contain a predicted C‐terminal TMD. Also, effectors BLR05 and BLR09 display a similar expression pattern during infection (Figure S2). Orthologs of BLR05 and BLR09 were recently identified in isolate SF5 that originates from Finland (Fletcher *et al*., 2019). The full‐length LsNAC069 protein interacts with the BLR05, BLR08, BLR09 and BLN04 effectors in the Y2H system, but not with BLN03 that only interacts with truncated forms of LsNAC069 lacking the NAC domain. The C‐terminus of LsNAC069 is formed by a TMD that is crucial for effector‐interaction. In contrast, the NAC domain and part of the variable domain are dispensable for this (Figure 4). The presence of TMDs raised the possibility that the effectors interact with their targets in or at one of the many membranes within plant cells similar to StNTP1/2 and their targeting effector Pi03192 (McLellan *et al*., 2013). Initially effectors and targets were transiently expressed separately as N‐ or C‐terminal fluorophore fusion proteins in *N. benthamiana*. Only N‐terminal fusions of BLR05, BLR08, BLR09 and LsNAC069 could be successfully visualized. BLR05, BLR09 and LsNAC069 predominantly localized at the ER membrane, although BLR05 was also found in Golgi bodies (Figure 2). Co‐expression of BLR05 and BLR09 with a truncated variant of LsNAC069, LsNAC069^ΔNAC^ that is more stable, confirmed their colocalization at the ER membrane (Figures 5 and S10). Transient expression of fluorescent BLR05 and BLR09 fusion proteins in lettuce showed a similar localization as observed in *N. benthamiana* (Figure S4). Yet, low protein levels of LsNAC069 in lettuce forced us to continue experiments in *N. benthamiana*.

Ultimately, co‐IP confirmed interaction of HA‐tagged BLR05 and BLR09 with CFP‐LsNAC069^ΔNAC^. In contrast, LsNAC069^174‐467^, that lacks the C‐terminal TMD, was not able to interact with the effectors in Y2H assays, nor in co‐IP. These results emphasize the importance of the C‐terminus and TMD of LsNAC069 for effector‐interaction.

BLR08 fusion protein was mainly detected in irregularly sized ring‐like structures, suggesting that overexpression of the effector induced cell stress (Jia *et al*., 2013). In addition, we were not able to confirm BLR08 as interactor of LsNAC069^ΔNAC^ by co‐IP *in planta*. We, therefore, did not further proceed with experiments on BLR08, and focused on the confirmed interactors BLR05 and BLR09 (Figure S3).

LsNAC069, BLR05 and BLR09 can be classified as tail‐anchored proteins (Borgese and Fasana, 2011; Shao and Hegde, 2011). Tail‐anchored proteins are inserted post‐translationally into the membrane. Proteins with a moderately hydrophobic TMD may insert via an unassisted pathway, whereas those with a strongly hydrophobic TMD require assistance from chaperones preventing aggregation of the hydrophobic domains (Brambillasca *et al*., 2006; Borgese and Fasana, 2011). Most likely, chaperones interact with LsNAC069 and *B. lactucae* effectors to guide ER membrane integration in plant cells. After the identification of TRC40 in mammalian cells and the Guided Entry of tail‐anchored proteins 3 (Get3) system in yeast cells (Borgese and Fasana, 2011), candidates for guiding membrane insertion of tail‐anchored proteins in plants are being elucidated (Maestre‐Reyna *et al*., 2017). The Arabidopsis genome encodes three homologous genes of the main components of the GET pathway, which have been demonstrated to function in the insertion of the tail‐anchored SYP72 into ER membranes (Srivastava *et al*., 2017). At this point, we can only speculate if translocated BLR05 and BLR09 need to be reintegrated into the ER membrane by the plants GET system or if they might enter the plant cell as vesicles able to fuse to the ER. Ultrastructural analyses have found very small vesicles and ER structures close to the EHM (Grouffaud *et al*., 2010; O'Connell and Panstruga, 2006). We can also imagine fusion of oomycete secreted vesicles containing tail‐anchored effectors with the EHM. Further transfer to the ER might be possible via EHM‐ER contact sides. The plasma membrane, which is the origin of the EHM, has been confirmed to interact with the ER through plasma membrane‐ER contact sites in mammalian and plant systems (Chang *et al*., 2013; Phillips and Voeltz, 2016; Pérez‐Sancho *et al*., 2016). The exact effector translocation mechanism clearly needs further investigation, in particular for membrane‐anchored effectors.

The Y2H system is generally considered unsuitable for detection of protein–protein interactions between integral membrane proteins. Membrane proteins with nuclear localization signals fused to the GAL4 DBD or AD are assumed to be unable to accumulate in the yeast nucleus or fold incorrectly (Thaminy *et al*., 2004). Yet, we identify multiple targets for tail‐anchored *B. lactucae* effectors, indicating that these effectors localize to the nucleus in yeast cells as GAL4‐DBD fusion proteins (Figures 1 and 3). Peculiarly, also Get3 recognition of TMDs of tail‐anchored proteins was demonstrated with Y2H (Schuldiner *et al*., 2008). A plausible explanation might be that some tail‐anchored proteins can be inserted into the inner nuclear membrane via the GET system (Laba *et al*., 2014). Although the GET system is associated with the ER membrane, these membranes are continuous with the nuclear inner and outer membranes, therefore passive diffusion can lead to low numbers of GET complexes in the inner nuclear membrane (Laba *et al*., 2014).

### Tail‐anchored NACs are conserved targets of pathogen effectors

Analysis of the NAC TF family in lettuce identified seven putative tail‐anchored NACs, which are grouped in subfamily 9 of the phylogenetic tree that encompasses a total of 94 lettuce NACs. In Arabidopsis, at least 14 tail‐anchored NACs are present (Kim *et al*., 2007a; Klein *et al*., 2012; Yang *et al*., 2014; Liang *et al*., 2015; Zhao *et al*., 2016), and 10 were found in potato (Singh *et al*., 2013). Phylogenetic analysis showed that LsNAC069 is closely related to StNTP2, ANAC013, ANAC016 and ANAC017 (Figure 3). *Bremia lactucae* effectors also interacted strongly with these potato and Arabidopsis NACs in targeted Y2H assays. Furthermore, the more distantly related proteins StNTP1 and ANAC116 also showed strong interactions with the effectors. For *Pseudomonas syringae*, it is known that HopD1, a type III effector, interacts with Arabidopsis NTL9 (ANAC116) at the ER, thereby suppressing an ETI response (Block *et al*., 2014). It has been shown that NTL9 is targeted by RxLR effector HaRXLL73 of the Arabidopsis downy mildew *Hyaloperonospora arabidopsidis* (Weßling *et al*., 2014). Also, an NTL9 T‐DNA insertion line displayed increased susceptibility to *H. arabidopsidis* (Mukhtar *et al*., 2011). NTL9 is found in group C of the tail‐anchored NAC tree, close to StNTP1 (Figure 3b), and in Y2H assays it was a strong interactor for all *B. lactucae* effectors and the *P. infestans* effector Pi03192 (Figure 3c). Our results demonstrate that ER‐associated tail‐anchored NACs form a conserved hub in multiple plant species and are targeted by a variety of pathogens.

### *Bremia lactucae* effectors inhibit stress*‐*induced relocalization of LsNAC069

Activation of tail‐anchored TF including LsNAC069 is thought to be dependent on regulated intramembrane proteolysis resulting in release of the TF from the membrane (Seo *et al*., 2008). Four classes of plant proteases mediate proteolysis within membranes: rhomboid serine proteases, site‐2 metalloproteases, and aspartyl proteases of the presenilin/γ‐secretases and signal peptide peptidases type. We observed that relocalization of CFP‐LsNAC069^ΔNAC^ was reduced in the presence of TPCK (Figure 6). TPCK is a broad‐spectrum inhibitor of chymotrypsin‐like serine proteases and some cysteine proteases (Bond and Butler, 1987). This makes the family of intramembrane rhomboid proteases a likely candidate for cleavage of LsNAC069 as it is the only intramembrane serine protease family (Weihofen and Martoglio, 2003; Adam, 2013). Two mechanisms have been proposed to govern substrate recognition by rhomboid proteases that are not mutually exclusive (Freeman, 2014). Multiple intramembrane protease substrates contain helix‐destabilizing residues, such as serine, glycine and proline (Cheng Li and Deber, 1994; Strisovsky *et al*., 2009), in their transmembrane region. This results in an intrinsically unstable TMD that may unwind in the aqueous environment within the protease to expose the cleavage site (Moin and Urban, 2012). Indeed, LsNAC069 contains helix‐destabilizing residues in its predicted TMD increasing the likelihood that it is an intramembrane protease substrate. Alternatively, rhomboid substrate specificity may be determined by recognition of a specific amino acid motif surrounding the cleavage site (Strisovsky *et al*., 2009). So far, only the recognition motif for a bacterial rhomboid protease has been determined (Strisovsky *et al*., 2009), and differences in rhomboid protease recognition motifs within and between species are expected to occur (Freeman, 2014). Unfortunately, this restricts extrapolation of the recognition motif to potential rhomboid protease substrates, including LsNAC069, in plants.

We found that treatment of *N. benthamiana* cells with *P. capsici* CF triggers relocalization of CFP‐LsNAC069^ΔNAC^ to the nucleus. *Phytophthora capsici* CF likely contains a mix of PAMPs, as was found previously for *P. infestans* CF that triggers upregulation of multiple markers of PTI (McLellan *et al*., 2013). However, the signaling pathways that bridge the gap between perception of triggers and execution of regulated intramembrane proteolysis are still poorly understood. We observed that nuclear accumulation of CFP‐LsNAC069^ΔNAC^ induced by CF was significantly reduced upon co‐expression of HA‐BLR05, and strongly reduced upon the combined expression of HA‐BLR05 and HA‐BLR09.

Furthermore, we decided to include PEG treatment into the relocalization experiment to gain insight of drought stress effects on ER‐to‐nucleus relocalization of LsNAC069. PEG is an osmolyte, which induces drought stress‐like responses in plants. Unexpectedly, PEG was a stronger inducer of CFP‐LsNAC069^ΔNAC^ relocalization than CF (Figure 6d). Additionally, effectors BLR05 and BLR09 were able to inhibit PEG‐induced CFP‐LsNAC069^ΔNAC^ relocalization. Overall, results indicate that CF‐ and PEG‐induced relocalization rely on a conserved mechanism that is triggered by different stress responses, for example, osmotic‐, drought‐ and plant immunity‐related stress.

### Unexpected phenotypes of *LsNAC069*‐silenced lettuce lines

Stable transformants expressing *LsNAC069* hpRNA were generated to evaluate the role of LsNAC069 in disease susceptibility (Figure 7). *LsNAC069‐*silenced plants did not display reproducibly altered susceptibility phenotypes to *B. lactucae* infection. We expected that LsNAC069 would contribute to disease resistance and silencing would enhance disease susceptibility as *StNTP1* or *StNTP2* silencing led to enhanced susceptibility to *P. infestans* (McLellan *et al*., 2013). The high level of susceptibility observed in the parental line *L. sativa* cv. Wendell may have obscured an enhanced susceptibility phenotype following *LsNAC069* silencing. Furthermore, considering the vast size of the NAC family in lettuce, it could well be that other redundant NAC family members are taking over the function of LsNAC069.

Even though no significant change in susceptibility to *B. lactucae* was observed in *LsNAC069* silenced lines, we observed a significantly increased resistance against *P. cichorii*, the causal agent of midrib rot in lettuce (Pauwelyn *et al*., 2011). Although our data indicate that LsNAC069 plays a role in susceptibility to this necrotrophic pathogen, it remains to be determined if interactions between LsNAC069 and effectors from *P. cichorii* occur during pathogen infection, or if silencing of LsNAC069 enhances resistance through systemic changes in the physiology of the silenced lettuce lines.

During propagation of homozygous *LsNAC069* silenced lines, we observed a decreased wilting response for the silenced lines. Indeed, phenotyping for drought stress‐treated plants revealed significant lower wilting of *LsNAC069* silenced lines in comparison to wild‐type plants (Figure 7), confirming the involvement of *LsNAC069* in drought stress adaptation. A variety of NAC TFs have been shown to be involved in drought stress, for example, SNAC3 in rice (Fang *et al*., 2015), ANAC019, ANAC055 and ATAF‐1 (Nuruzzaman *et al*., 2013). Interestingly, some of them are also involved in plant defense against necrotrophic pathogens (Nuruzzaman *et al*., 2013). Also, the Arabidopsis loss‐of‐function *ocp3* (homeobox TF) mutant exhibits both drought resistance and enhanced disease resistance to necrotrophic fungal pathogens, showing how closely co‐regulated drought stress and immunity can be (Ramírez *et al*., 2009). Generally, the interplay between host, pathogen and environment is famously known as the ‘disease triangle’ (Stevens, 1960). Among environmental factors, periods of high atmospheric humidity have repeatedly been shown to promote disease outbreaks in plants, suggesting that the availability of water is crucial for pathogenesis (Xin *et al*., 2016; Aung *et al*., 2018). A variety of pathogenic Pseudomonas strains create an aqueous habitat in the leaf apoplast during the early infection period. Based on our results on reduced drought stress and *P. cichorii* infection in *LsNAC069* silenced lines and PEG‐induced relocalization, we hypothesize that LsNAC069 is involved in negative regulation of *P. cichorii*‐induced water soaking, but in positive regulation of drought stress resistance. How LsNAC069 is involved in these regulation mechanisms needs to be further elucidated, and might help us to gain understanding of the interplay of plant water regulation mechanisms and its effect on infection by different microbial pathogens.

## Experimental procedures

### Plant growth and drought stress assay

Lettuce seed germination and *B. lactucae* disease assays were performed under short‐day growth conditions [9 h of light (100 μE m^−2^ sec^−1^)] at 16°C. *Nicotiana benthamiana* plants and germinated lettuce seedlings were grown under long‐day conditions (16 h of light, 70% humidity) at 21°C.

Drought stress experiments were performed under long‐day conditions with 4‐week‐old plants. Two weeks before the start of the drought stress experiment, each plant was given 150 ml of water per day. From time point 0 onwards, the control group continued to receive water, whereas water was withheld from the drought stress group. Phenotypes were documented, and pictures used to measure the total visible shoot surface in cm^2^ with image J Fiji.

### Lettuce cDNA library construction

*Lactuca sativa* cv. Olof seedlings were sprayed with water, 0.1 mg ml^−1^ benzothiadiazole (BTH) solution, *B. lactucae* race Bl:24 (compatible interaction) or isolate F703 (incompatible interaction) spore suspension. RNA was extracted using phenol/chloroform extraction at 24 h after BTH treatment and 3 days after infection; 2 mg of RNA (0.5 mg per treatments) was used to construct a three‐frame uncut cDNA library in prey vector pDEST22 by Invitrogen Custom Services (Invitrogen, Carlsbad, CA, USA).

### Vector construction

Illumina‐based RNAseq was performed on mRNA derived from *B. lactucae* spores and *B. lactucae*‐infected lettuce seedlings (Giesbers *et al*., 2017). Novel effector candidates were discovered by analysis of the secretome (Giesbers *et al*., 2017) with a Perl script using regular expressions for RXLR‐like, dEER and LXLFLAK motifs. Secondly, homology search of the secretome against a database composed of known effector sequences was conducted, and coding sequences were PCR amplified from BL:24 cDNA (without signal peptide) as described in Pelgrom *et al*. (2019). Generation of pENTR/D‐TOPO clones with *B. lactucae* effectors BLN03^22‐169^, BLN04^24‐147^, BLR05^22‐97^, BLR08^30‐135^ and BLR09^23‐112^ was accomplished according to Stassen *et al*. (2013). *Bremia lactucae* effector entry clones, pDONR221‐Pi03192 and pDONR201‐PiAVR2 (McLellan *et al*., 2013), were recombined with pDEST32 to generate GAL4 DNA‐binding domain (DBD) fusion proteins.

All vector constructs were generated using Gateway cloning. The *LsNAC069* coding sequence and truncated variants were cloned into a modified pGemTEasy (pGemTEasy^mod^) vector containing the pDONR201 Gateway recombination site. LsNAC069, StNTP1, StNTP2 (McLellan *et al*., 2013), ANAC013, ANAC017, ANAC053 and ANAC078 (De Clercq *et al*., 2013) were transferred to pDEST22. pDEST22‐ANAC016, pDEST22‐ANAC086 and pDEST22‐CBNAC/NTL9 plasmids were obtained from an Arabidopsis TF Y1H library (Pruneda‐Paz *et al*., 2014).

Entry clones of *B. lactucae* effectors and their targets were recombined using LR clonase with pB7WGY2, pB7WGC2 and pB7WGR2 (Karimi *et al*., 2005) to create C‐ and N‐terminal YFP, CFP and RFP fusion proteins, with pEarleyGate201 to generate HA fusion proteins.

Two *LsNAC069* gene fragments of 342 and 335 bp were amplified (primer, Table S3) and cloned in the pHELLSGATE12 vector (Helliwell and Waterhouse, 2003) to generate LsNAC069 hairpin(hp)RNA constructs.

### Yeast‐two‐hybrid screens

All yeast transformations were performed using the TE/LiAc method (Schiestl and Gietz, 1989). The lettuce cDNA library was transformed in yeast strain Y8800 (MATa trp1−901 leu2−3,112 ura3−52 his3− 200 gal4Δ gal80Δ cyh2R GAL1::HIS3@LYS2 GAL2::ADE2 GAL7::LacZ@met2), whereas *B. lactucae* effector constructs were transformed in yeast strain Y8930 (genotype MATα trp1−901 leu2−3,112 ura3−52 his3−200 gal4Δ gal80Δ cyh2R GAL1::HIS3@LYS2 GAL2::ADE2 GAL7::LacZ@met2). Effector strains auto‐activating the *HIS3* reporter were discarded.

Library Y2H screens were performed using the mating method (Fromont‐Racine *et al*., 2002). Successful bait–prey interactions were identified by selection of diploid yeast on ‐Leu ‐Trp ‐His plates. Prey vector inserts were amplified from yeast by colony polymerase chain reaction (PCR; Amberg *et al*., 2006). Purified PCR products were Sanger sequenced and prey identity determined by BLASTn against our lettuce transcriptome (Pelgrom *et al*., 2019) and the *L. sativa* cv. Salinas reference genome v8 (Reyes‐Chin‐Wo *et al*., 2017). Out‐of‐frame proteins and proteins with an early stop codon were discarded. For further verification, bait plasmid and prey plasmid isolated from a representative yeast colony were co‐transformed in yeast strain Y8930 and reporter gene activation was assessed. Targeted Y2H assays were performed by co‐transformation of bait and prey plasmid in yeast strain Y8930.

### Transient expression in *Nicotiana benthamiana* and lettuce

Constructs for transient expression in lettuce and *N. benthamiana* were transformed in *Agrobacterium tumefaciens* strain C58C1 (pGV2260). Transient expression by *Agrobacterium* infiltration of lettuce was accomplished as described in Pelgrom *et al*. (2019). Infiltration of *N. benthamiana* for transient expression via *Agrobacterium* was accomplished as described by Li (2011) with a final OD_600_ of 0.4. For relocalization experiments, *Agrobacterium* containing LsNAC069^ΔNAC^ construct at OD_600_ of 0.4 was mixed with *Agrobacterium* strains containing empty vector or effectors to a final OD_600_ of 1.

### Relocalization assay

One day after infiltration of *N. benthamiana* leaves with *Agrobacterium*, infiltrated leaf sections were cut out and incubated in MS medium (‐vitamins) and the corresponding treatments for 16 h in the dark at RT. Proteasome inhibitor MG‐132 (#474790, Bio‐Connect, Huissen, the Netherlands) and *N*‐p‐tosyl‐l‐phenylalanine chloromethyl ketone (TPCK; #T4376, Sigma, Darmstadt, Germany) were prepared in dimethylsulfoxide and added to a final concentration of 25 μm each. Polyethylene glycol‐6000 (PEG; #8.07491, Sigma) was dissolved in MS medium and added to the final concentration of 5% (v/v) in relocalization assays. Culture filtrate for treatment was prepared by cutting V8 plates [20% (v/v) V8 juice, 1.5% (w/v) agar, 35 mm CaCO_3_] containing *P. capsici* (Pc LT3112) mycelium in squares and submerging these in water. After 2 days, water was renewed and the plate transferred to 4°C for 1 h. Supernatant was filtered through a 0.2‐μm syringe filter. All experiments were accomplished in at least three independent replicates with a total number of *n* > 13. Zeiss Zen 2.3 lite was used for the relative quantification of fluorescence intensity in nuclei (integral of the circular area), followed by statistical validation and diagram preparation with python.

### Confocal imaging

Confocal imaging was performed on Zeiss 700 (Zeiss, Germany) using the 63 × objective with oil emersion (Objective Plan‐Apochromat 63 ×/1.40 Oil DIC). CFP, YFP and RFP were excited at 405, 488 and 555 nm, respectively. Emitted light of both CFP and YFP was captured using a 490–555 nm bandpass filter, and emitted light of RFP was captured using a 560‐nm‐long pass filter. Analysis of the images was performed with ZEN lite v2.3.

### Co‐immunoprecipitation and immunoblotting

Leaves were harvested 48 h after infiltration with Agrobacterium suspensions, frozen in liquid nitrogen and stored until further processing at −80°C. All the following steps were performed at 4°C. Leaves were ground and proteins extracted with lysis buffer [10 mm Tris‐HCl pH 7.6, 150 mm NaCl, 0.5 mm EDTA, 0.25% (v/v) Triton‐X 100, 0.25% (v/v) Tween‐20, 5 mm dithiothreitol, 4% (w/v) polyvinylpyrrolidone] supplemented with 2 × protease inhibitor cocktail without EDTA (#11873580001, Roche, Mannheim, Germany) for 1 h. Lysates were centrifuged for 15 min at 10 000 ***g***. Supernatant was mixed with 4 × Bolt LDS sample buffer (ThermoFisher Scientific, Carlsbad, CA, USA) and processed according to the manufacturer's instructions. Samples for co‐IP were further processed as recommended by manufacturers (GFP‐trap, Chromotek, Planegg‐Martinsried, Germany and μMACS HA, Miltenyi Biotec, Gladbach, Germany). Protein separation was accomplished with Bolt pre‐cast gradient gels 4–12% with Bolt Mes‐SDS running buffer (ThermoFisher Scientific). Western blot was accomplished for 50 min at 100 V to nitrocellulose membrane using Towbin buffer [25 mm Tris, 192 mm glycine, 20% (v/v) methanol] and detected with the anti‐GFP (ChromoTek) or anti‐HA (Miltenyi Biotec) HRP conjugated antibodies. Proteins were detected using SuperSignal West Pico chemiluminescent substrate (ThermoFisher Scientific) and documented with a CCD‐camera (ChemiDoc, Bio‐Rad, Veenendaal, the Netherlands).

### Stable silencing of *LsNAC069* in lettuce

pHELLSGATE12 constructs were transformed in *A. tumefaciens* strain GV2260MPI. *Lactuca sativa* cv. Wendell was transformed as described in Pileggi *et al*. (2001), and transformants were selected on kanamycin. Silencing efficiency and specificity were determined using quantitative reverse transcriptase‐polymerase chain reaction (qRT‐PCR) in T2 plants.

### Gene expression analysis

To determine the expression level of *B. lactucae* effectors and LsNAC069 during *B. lactucae* infection, qRT‐PCR was performed on cDNA of 4‐day‐old plants at 3 h, 1, 3 and 6 days after BL:24 infection (100 spores μl^−1^). Furthermore, LsNAC069 expression levels of hpRNA silenced lettuce plants were determined. Relative transcript levels were determined using SYBR Green PCR Master mix (ThermoFisher Scientific) and the ViiA7 Real‐Time PCR system (ThermoFisher Scientific). The primers used for qRT‐PCR are listed in Table S2.

### Disease assays

Leaf discs were punched from 4‐week‐old T2 plants, placed upside down on filter paper soaked with MilliQ water and spray‐inoculated with a spore suspension of *B. lactucae* race Bl:24 (40 spores μl^−1^). Trays with leaf discs were closed with a transparent lid and sealed with tape. The infection severity was determined at 11 days after infection by scoring the area of the leaf disc covered in sporangiophores.

*Pseudomonas cichorii* disease assays were performed on 5‐week‐old plants grown under long‐day conditions. The protocol was modified from Liu *et al*. (2015), and plants were infiltrated with a final concentration of OD_600_ = 0.0005 of *P. cichorii* strain AB121. Bacterial growth, measured as colony‐forming units, was determined at 0 and 48 h after infiltration.

### Bioinformatic analyses

*Lactuca sativa* cv. Salinas coding sequences (CDS) corresponding to genome v8 (Genome ID 28333) were downloaded from the CoGe platform (https://genomevolution.org/). Translated CDSs were searched using Hidden Markov Models for the presence of NAM (PF02365) domains with *E*‐value 1e^−4^ as the cut‐off. TMDs were predicted using TMHMM 2.0 (Sonnhammer *et al*., 1998; Krogh *et al*., 2001) and TOPCONS (Bernsel *et al*., 2009; Tsirigos *et al*., 2015).

All identified NAC genes were named. Gene names are composed of the prefix ‘*Ls*’ for *L. sativa* followed by NAC and a number. NAC genes were numbered according to their position on lettuce linkage groups 1–9 (Table S2).

Alignment of NAC proteins was performed using Clustal Omega (Sievers *et al*., 2014) with default parameters. Poorly aligned sequences were removed from the lettuce NAC protein alignment and sequences were realigned. Phylogenetic trees were constructed using MEGA 7.0 (Kumar *et al*., 2016) using the Neighbor‐Joining method and bootstrap values based on 1000 iterations. Pairwise gap deletion was used for construction of a phylogenetic tree from the lettuce NAC proteins alignment. Trees were visualized using iTOL software (Letunic and Bork, 2016).

## Conflict of interest

The authors declare that they have no conflict of interest.

## Data statement

Supporting information was made available in the supplemental figures and tables. Unprocessed images used in this study will be available upon request from the corresponding author.

## Supporting information


**Figure S1.** Alignment of yeast isolated prey inserts to LsNAC069.

**Figure S2.** Expression of LsNAC069 and effectors BLR05, BLR08, BLR09 and BLN04 during *Bremia lactucae* infection.

**Figure S3.** Subcellular localization of *Bremia lactucae* effector BLR08 in *Nicotiana benthamiana*.

**Figure S4.** *Bremia lactucae* effectors BLR05, BLR09 and CFP‐LsNAC069 predominantly label the ER membrane in lettuce.

**Figure S5.** Phylogenetic relationship of NAC proteins in lettuce.

**Figure S6.** Graphical representation of potato and Arabidopsis NACs used in Y2H.

**Figure S7.** Western blotting of CFP‐LsNAC069 and truncations.

**Figure S8.** MG132 has a stabilizing effect on the full LsNAC069.

**Figure S9.** LsNAC069 and effectors BLR05 and BLR09 localize to the ER.

**Figure S10.** Co‐expression of LsNAC069^ΔNAC^ with *Bremia lactucae* effectors and ER marker in *Nicotiana benthamiana*.

**Figure S11.** V8 control does not induce relocalization of CFP‐LsNAC069^ΔNAC^ to the nucleus.

**Figure S12.** Expression of proteins during translocation experiments.

**Figure S13.** *LsNAC091* transcript levels.

**Figure S14.** Nuclear accumulation of LsNAC069^ΔNAC^ induced by PEG is inhibited in the presence of *Bremia lactucae* effectors.

**Figure S15.** *LsNAC069* silencing reduces wilting effects during drought stress at 48 h but plants adjust to the wild‐type phenotype at 72 h.

**Table S1.** Targets identified in Y2H screening.

**Table S2.** Overview of lettuce NAM domain containing genes.

**Table S3.** Primers.
