# Supplementary figures and images for: Multiple downy mildew effectors target the stress‐related NAC transcription factor LsNAC069 in lettuce
Source: Plant J. 2019 Jul 4;99(6):1098–115. doi: 10.1111/tpj.14383 (PMC9545932; doi:10.1111/tpj.14383)

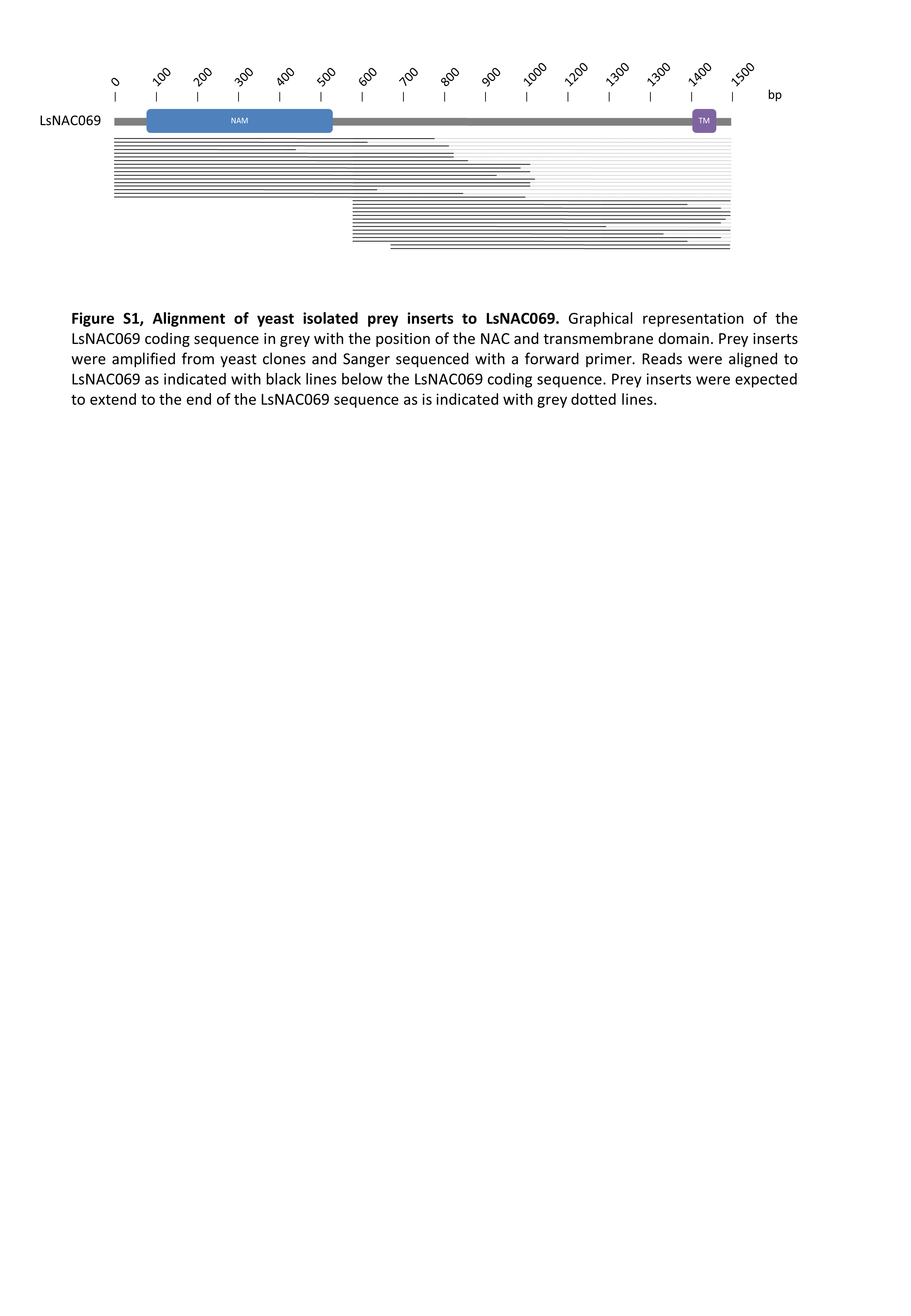

Supplement: Supplementary file 1 — Figure S1. Alignment of yeast isolated prey inserts to LsNAC069. [file TPJ-99-1098-s001.tif]

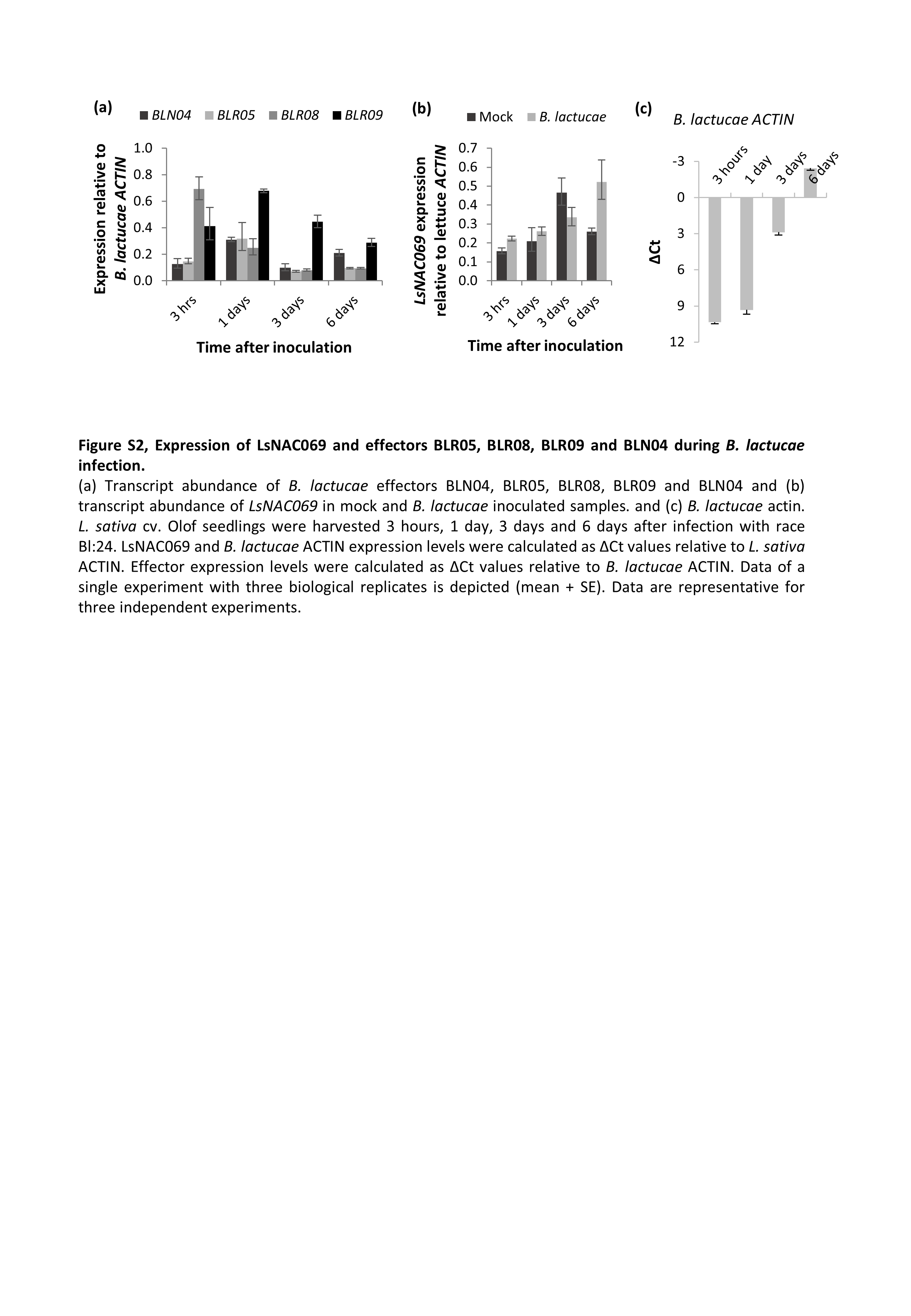

Supplement: Supplementary file 2 — Figure S2. Expression of LsNAC069 and effectors BLR05, BLR08, BLR09 and BLN04 during Bremia lactucae infection. [file TPJ-99-1098-s017.tif]

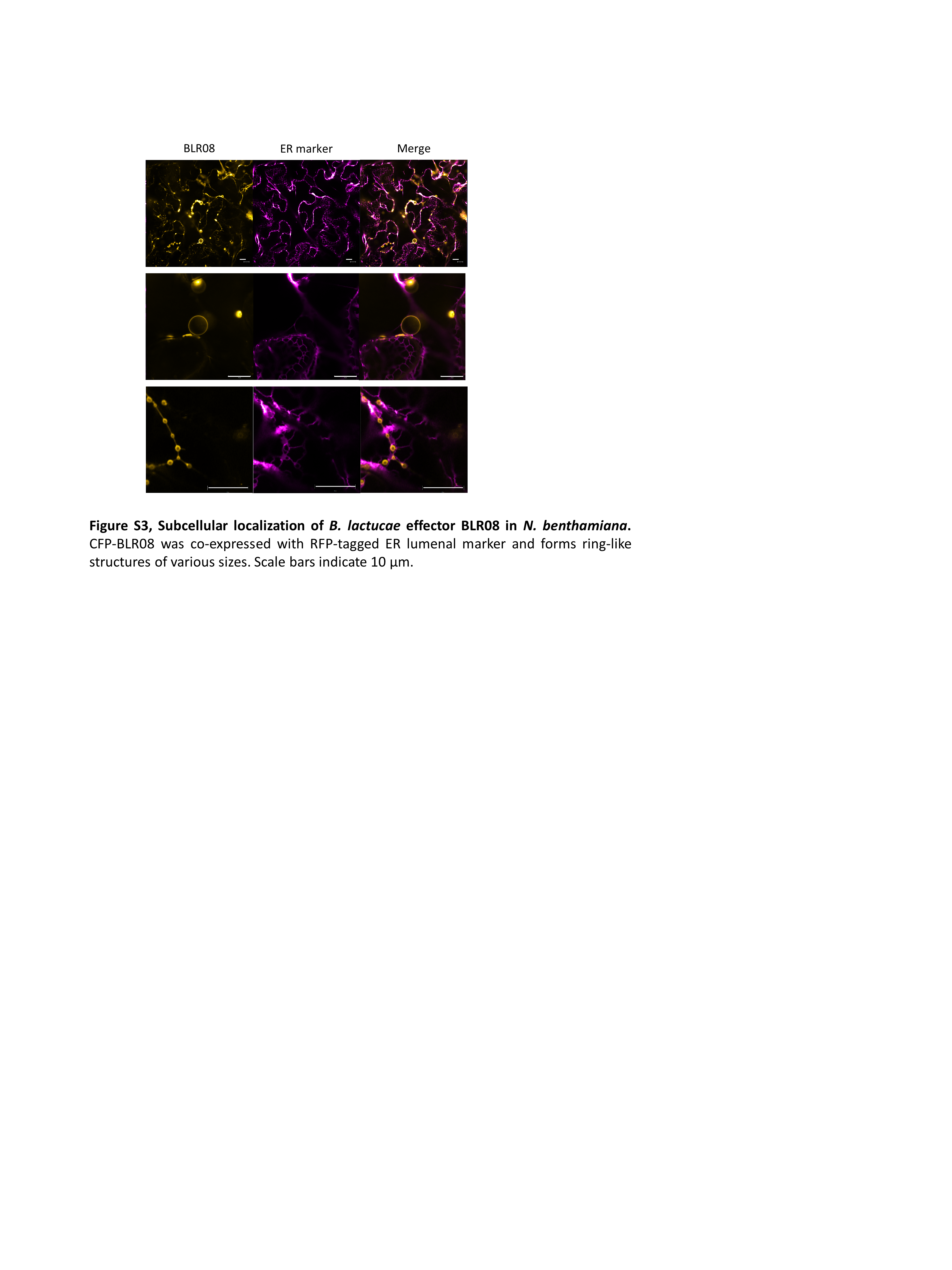

Supplement: Supplementary file 3 — Figure S3. Subcellular localization of Bremia lactucae effector BLR08 in Nicotiana benthamiana. [file TPJ-99-1098-s016.tif]

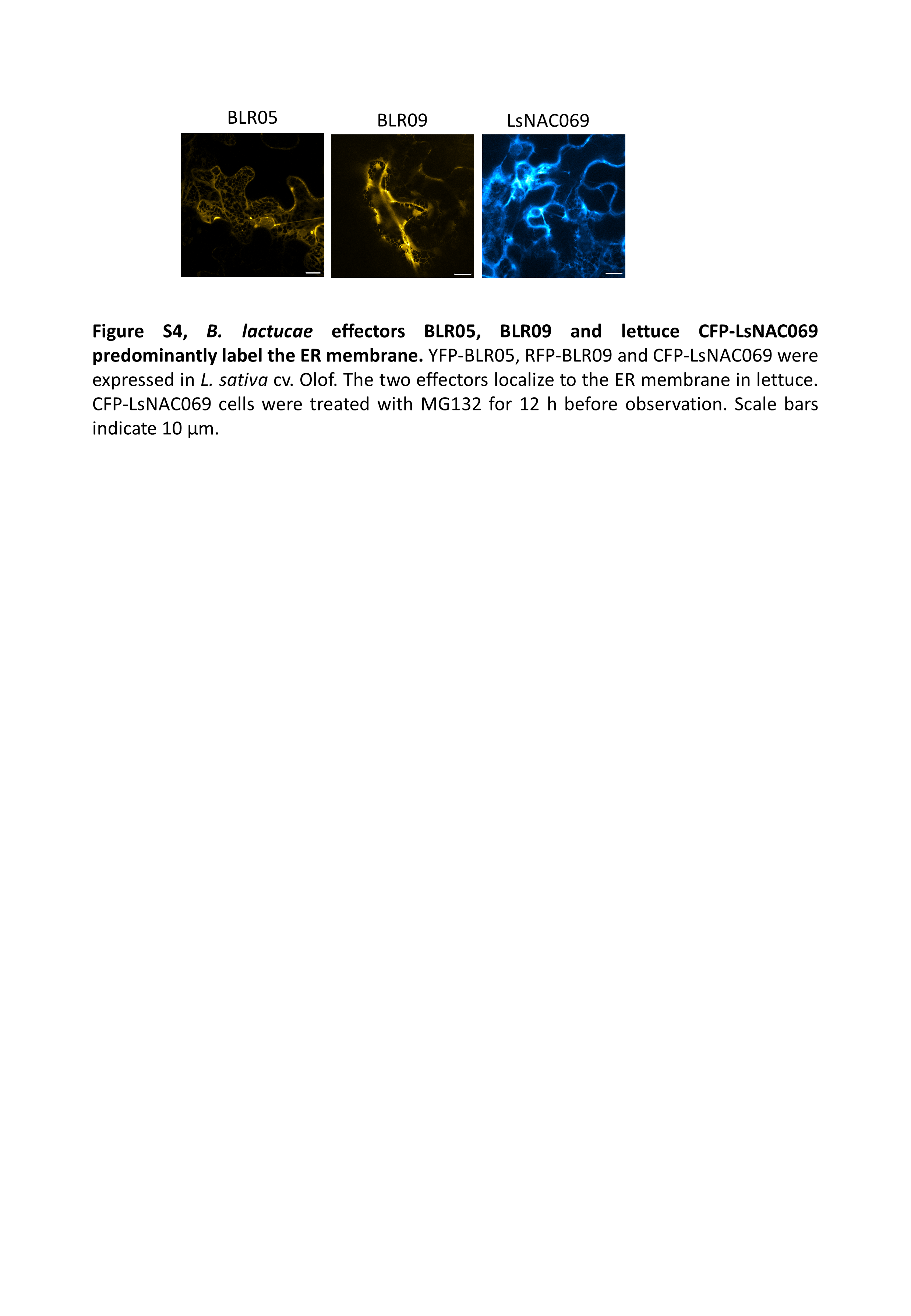

Supplement: Supplementary file 4 — Figure S4. Bremia lactucae effectors BLR05, BLR09 and CFP‐LsNAC069 predominantly label the ER membrane in lettuce. [file TPJ-99-1098-s018.tif]

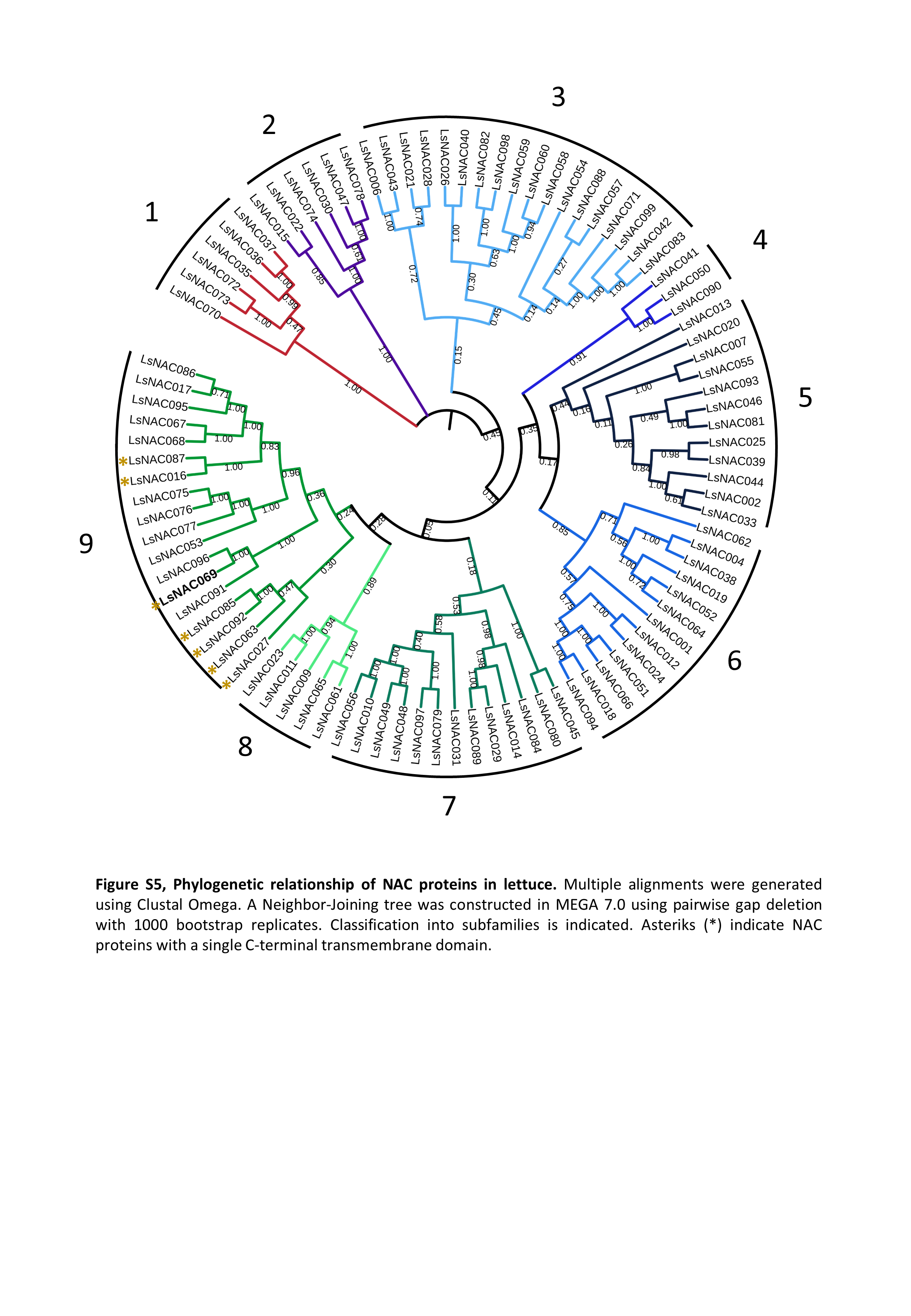

Supplement: Supplementary file 5 — Figure S5. Phylogenetic relationship of NAC proteins in lettuce. [file TPJ-99-1098-s019.tif]

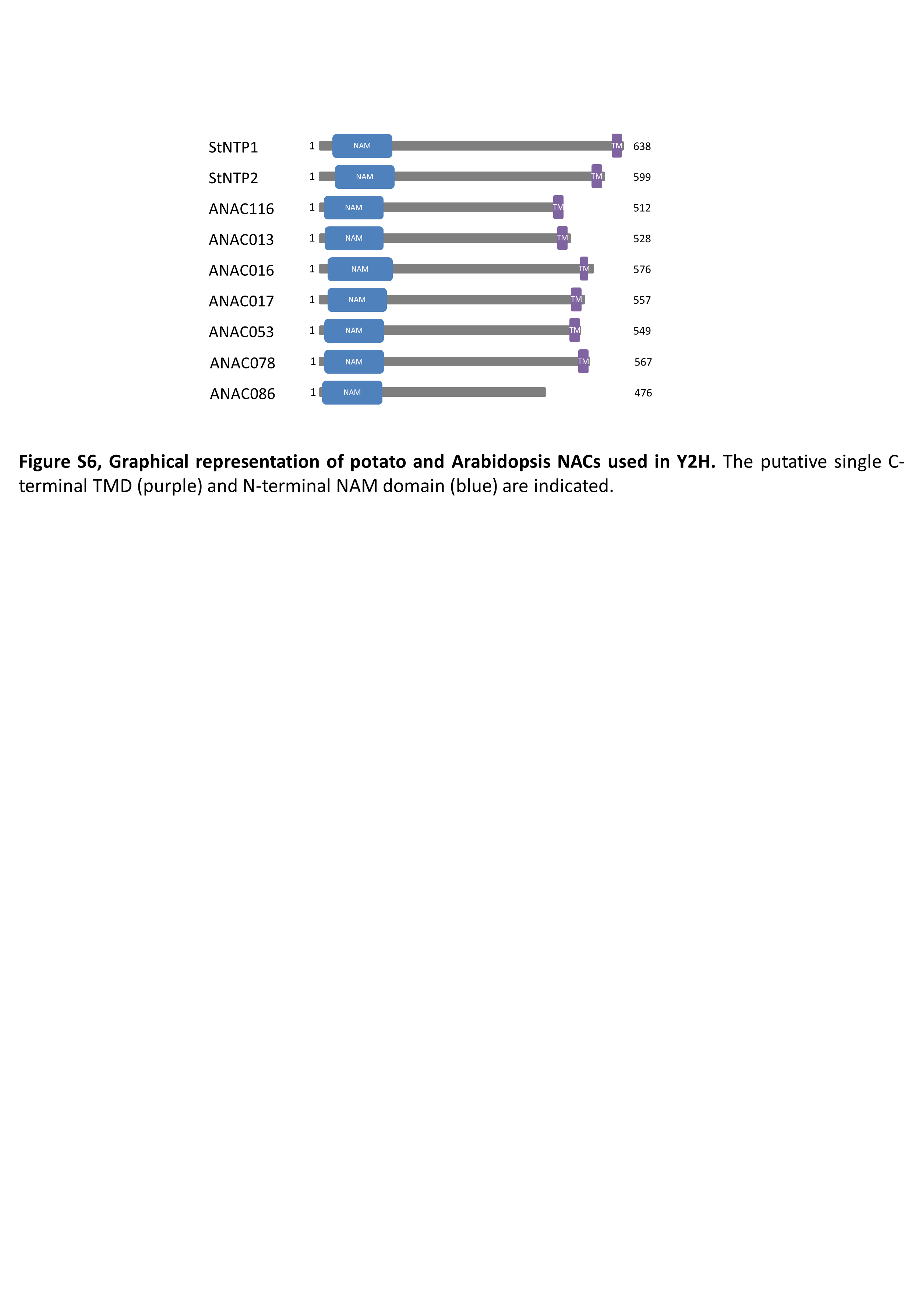

Supplement: Supplementary file 6 — Figure S6. Graphical representation of potato and Arabidopsis NACs used in Y2H. [file TPJ-99-1098-s002.tif]

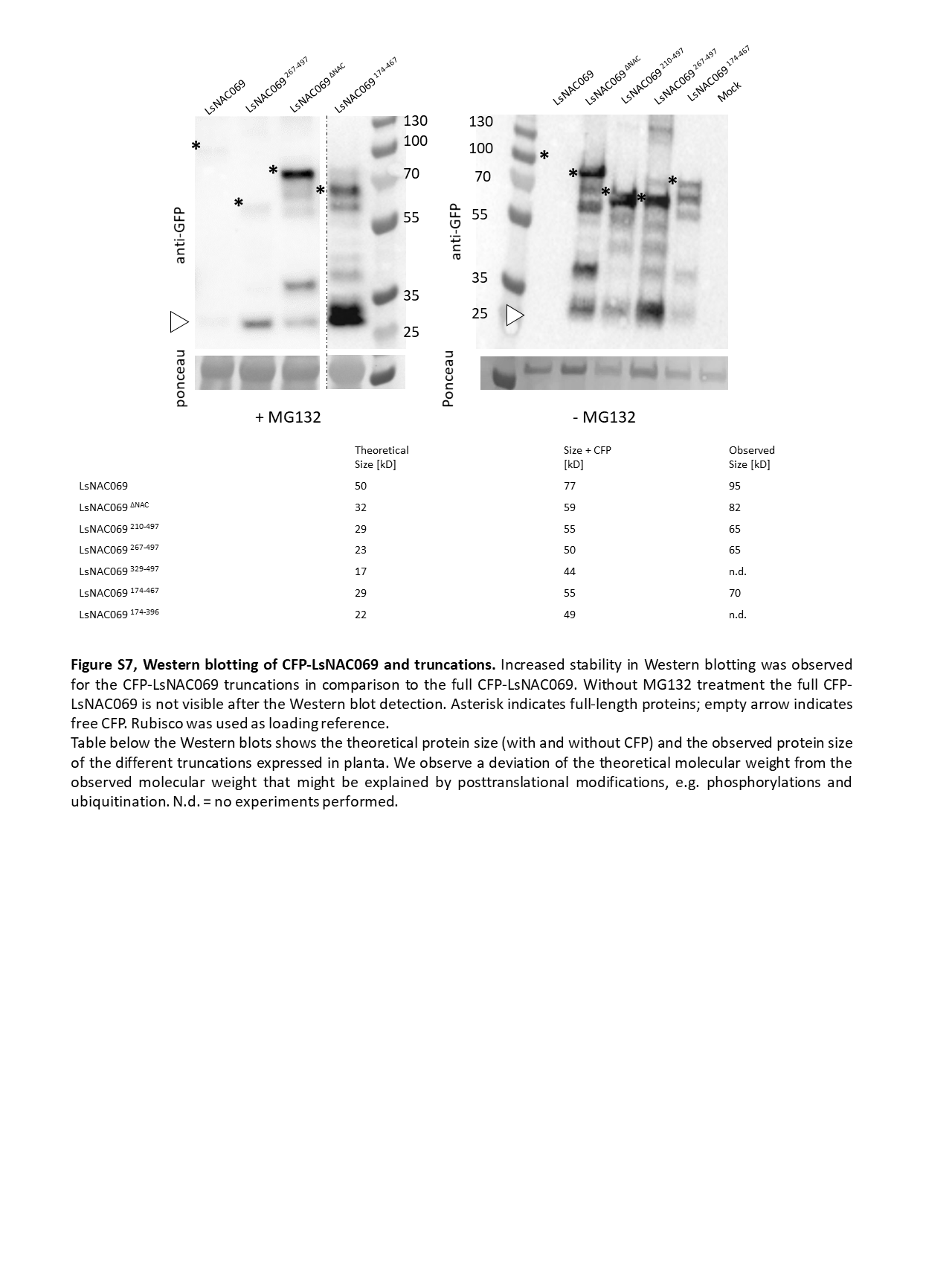

Supplement: Supplementary file 7 — Figure S7. Western blotting of CFP‐LsNAC069 and truncations. [file TPJ-99-1098-s003.tif]

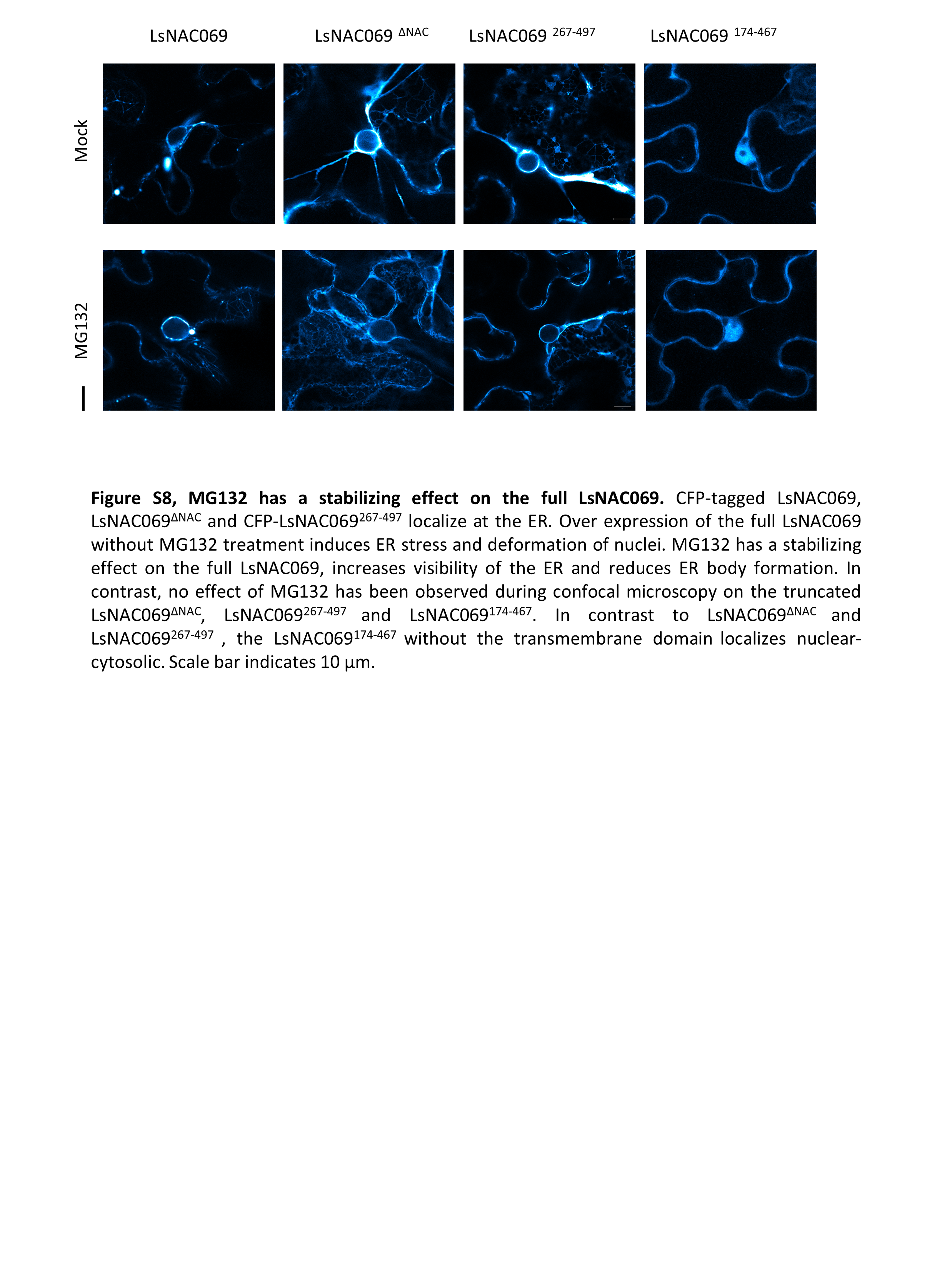

Supplement: Supplementary file 8 — Figure S8. MG132 has a stabilizing effect on the full LsNAC069. [file TPJ-99-1098-s004.tif]

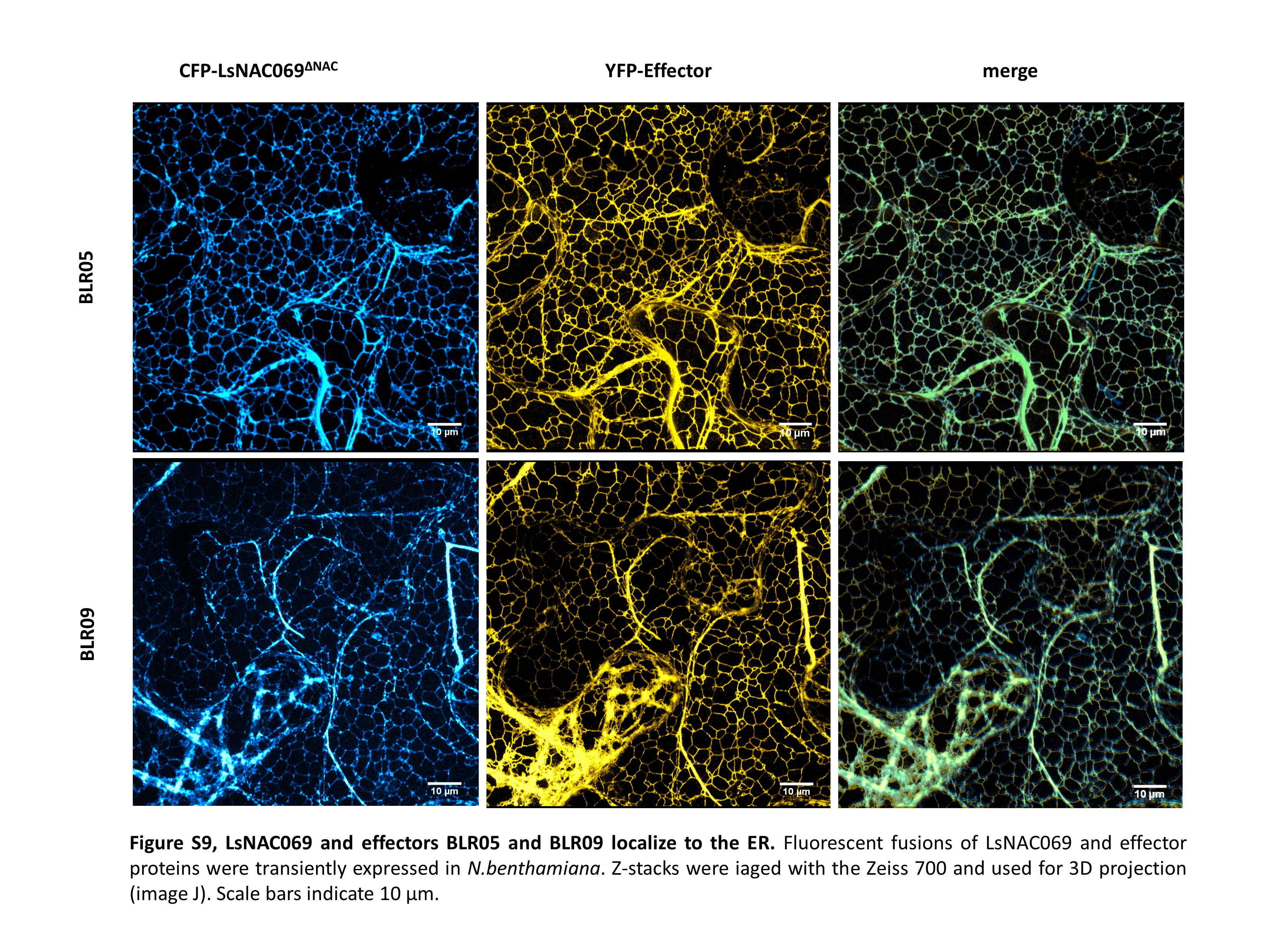

Supplement: Supplementary file 9 — Figure S9. LsNAC069 and effectors BLR05 and BLR09 localize to the ER. [file TPJ-99-1098-s005.tif]

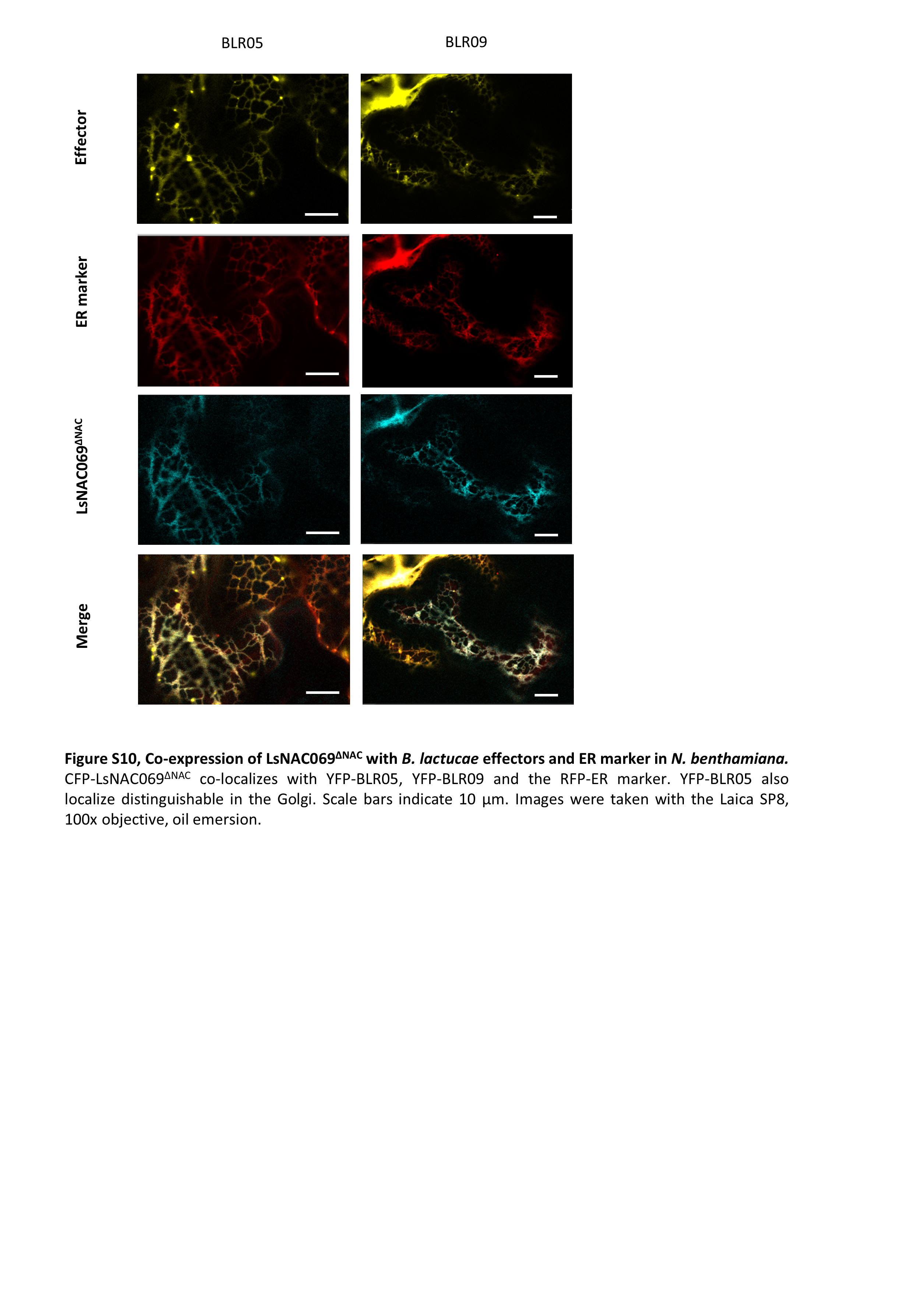

Supplement: Supplementary file 10 — Figure S10. Co‐expression of LsNAC069ΔNAC with Bremia lactucae effectors and ER marker in Nicotiana benthamiana. [file TPJ-99-1098-s006.tif]

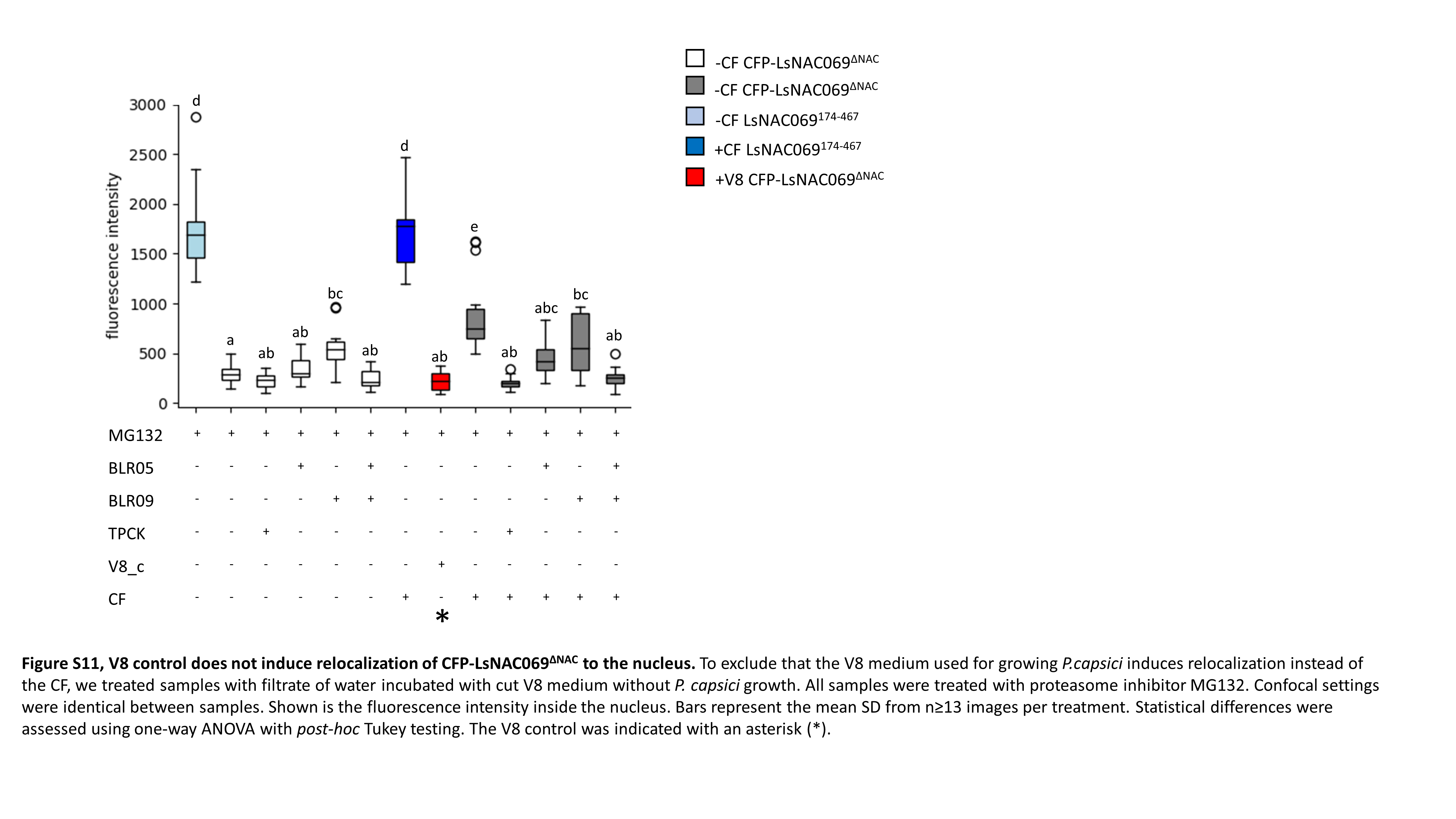

Supplement: Supplementary file 11 — Figure S11. V8 control does not induce relocalization of CFP‐LsNAC069ΔNAC to the nucleus. [file TPJ-99-1098-s007.tif]

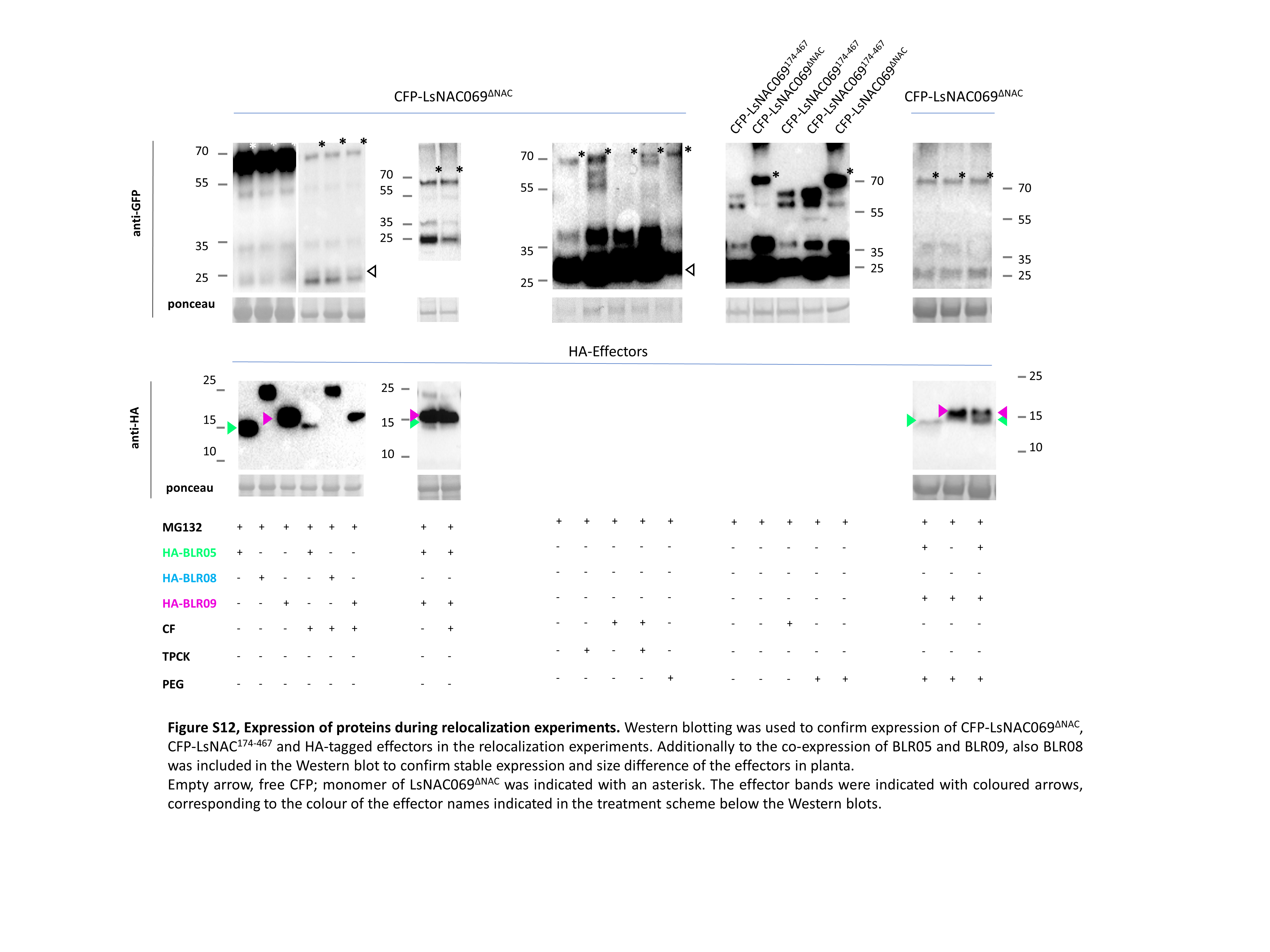

Supplement: Supplementary file 12 — Figure S12. Expression of proteins during translocation experiments. [file TPJ-99-1098-s008.tif]

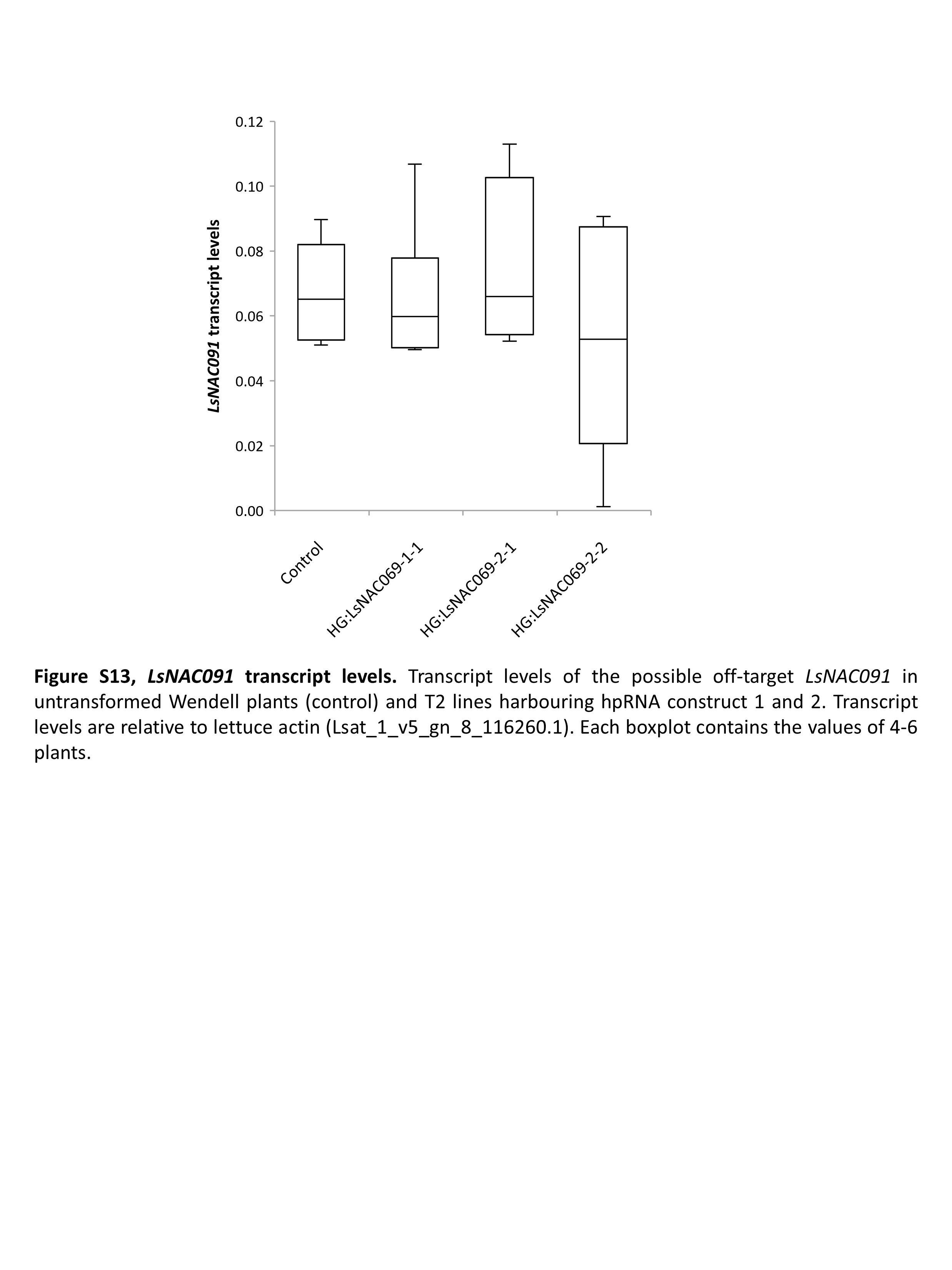

Supplement: Supplementary file 13 — Figure S13. LsNAC091 transcript levels. [file TPJ-99-1098-s009.tif]

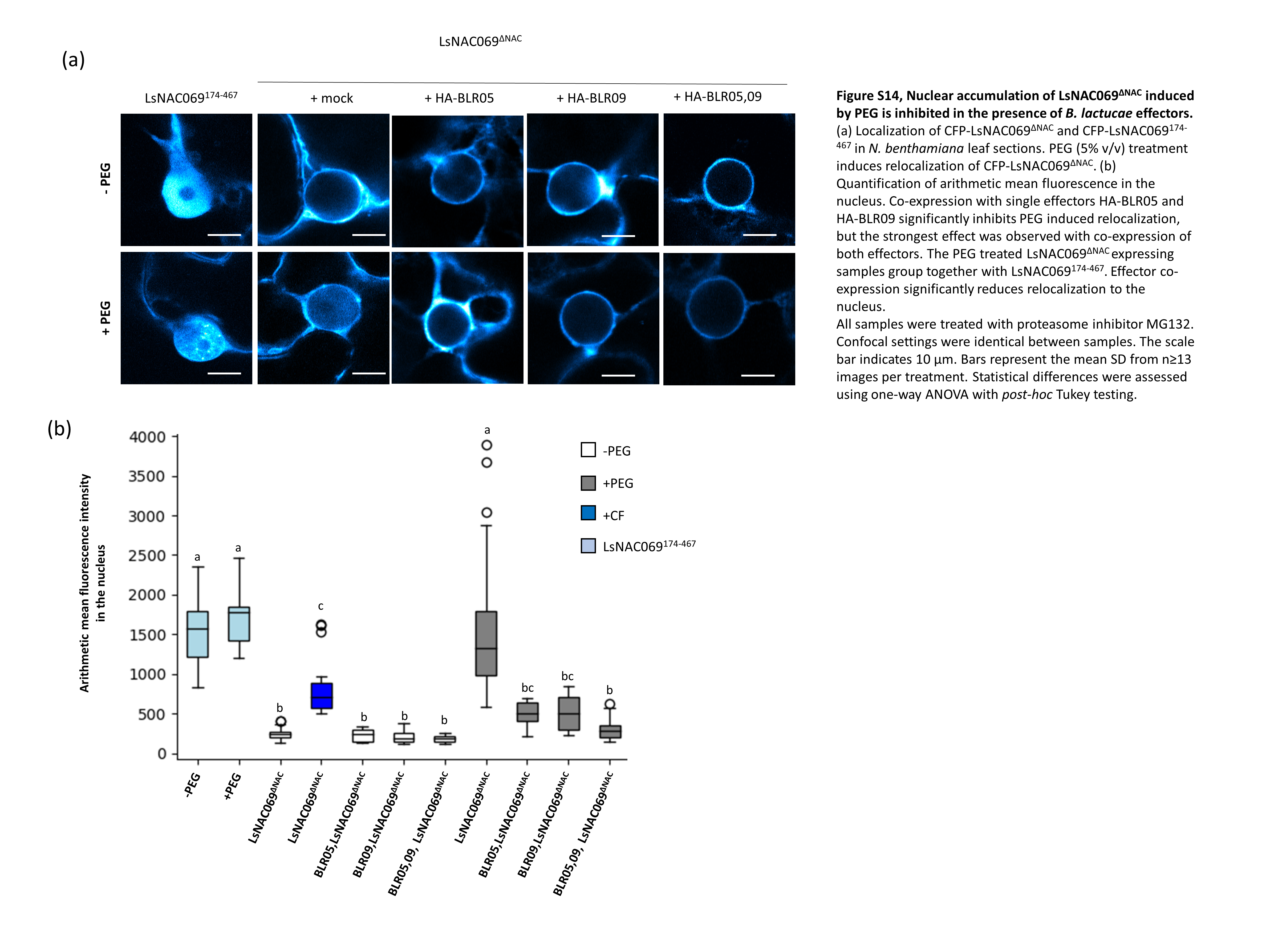

Supplement: Supplementary file 14 — Figure S14. Nuclear accumulation of LsNAC069ΔNAC induced by PEG is inhibited in the presence of Bremia lactucae effectors. [file TPJ-99-1098-s010.tif]

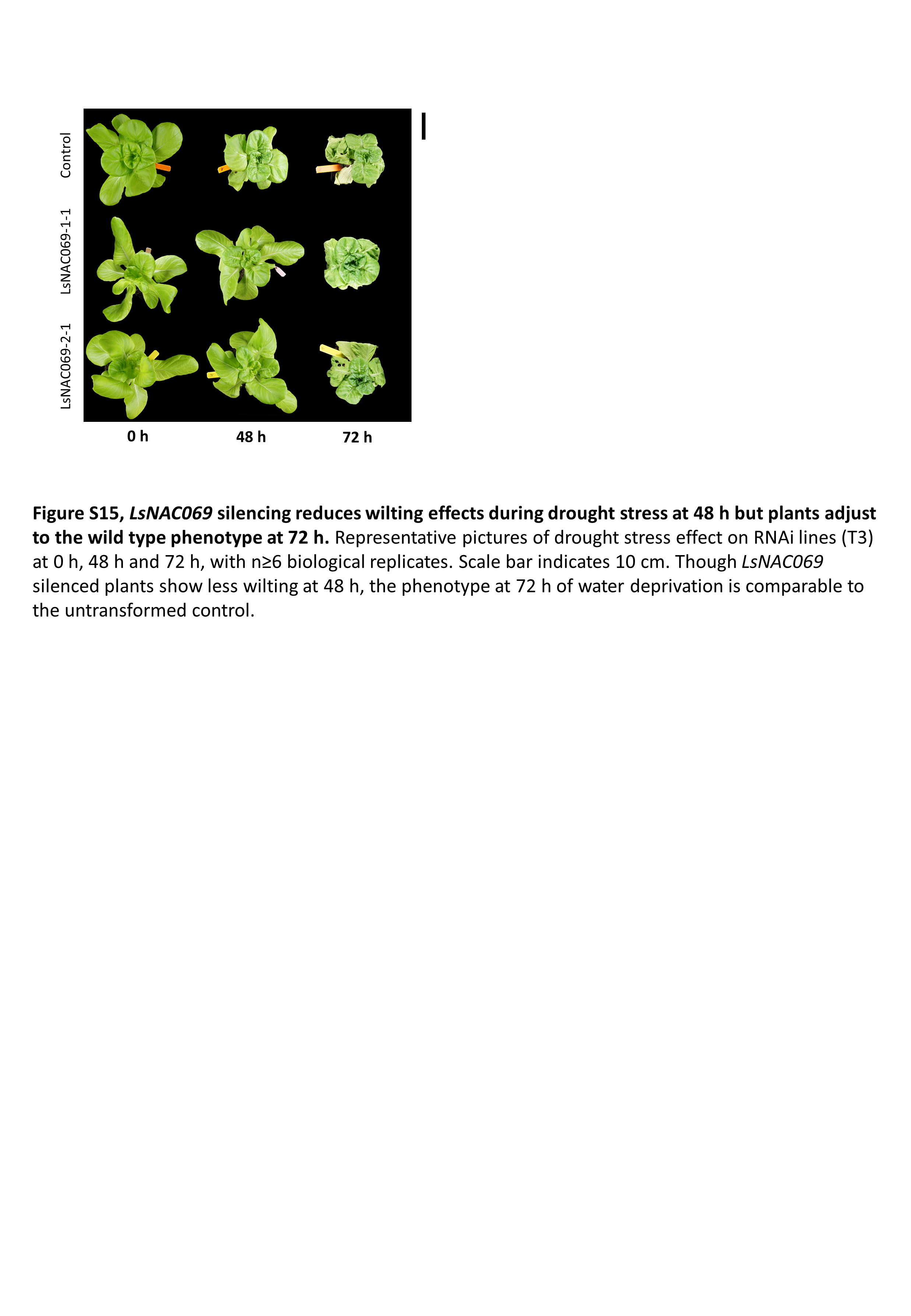

Supplement: Supplementary file 15 — Figure S15. LsNAC069 silencing reduces wilting effects during drought stress at 48 h but plants adjust to the wild‐type phenotype at 72 h. [file TPJ-99-1098-s011.tif]
