# Supplementary material for: Multiple downy mildew effectors target the stress‐related NAC transcription factor LsNAC069 in lettuce
Source: Plant J. 2019 Jul 4;99(6):1098–115. doi: 10.1111/tpj.14383 (PMC9545932; doi:10.1111/tpj.14383)
Supplement: Supplementary file 16 — Table S1. Targets identified in Y2H screening. [file TPJ-99-1098-s012.docx]

**Supplemental Table 1, Lettuce proteins identified in yeast-two-hybrid screens**

| Lactuca ID^1^ | Interacting effectors  (# colonies) | Length (aa) | Signal peptide ^2^ | Transmembrane domains ^3^ | Domains/ family ^4^ |
| --- | --- | --- | --- | --- | --- |
| Lsa002122.1 | BLN03 (6), BLN04 (13), BLR05 (29), BLR08 (25), BLR09 (31) | 296 | - | 129-147; 151-173; 217-239 | Reticulon domain |
| Lsa008464.1 | BLN04 (9), BLR05 (23), BLR08 (9), BLR09 (15) | 191 | 1-24 | 114-136 | - |
| Lsa040031.1 | BLN04 (4), BLR05 (14), BLR08 (6), BLR09 (7) | 497 | - | 467-486 | NAC domain |
| Lsa002329.1 | BLR05 (2) | 137 | - | 111-133 | Cytochrome b5-like heme/steroid binding domain |
| Lsa034832.1 | BLR05 (2) | 171 | - | 135-157 | - |
| Lsa039137.1 | BLR05 (3) | 279 | - | 111-133; 159-181; 248-270 | Uncharacterised protein family UPF0114 |
| Lsa001248.1 | BLR08 (2) | 682 | - | 522-544; 614-636 | Protein of unknown function DUF639 |
| Lsa020711.1 | BLR09 (2) | 596 | - | 568-590 | NAC domain |
| Lsa027896.1 | BLR09 (3) | 341 | - | - | AIG1-type guanine nucleotide-binding (G) domain |
| Lsa033367.1 | BLR09 (2) | 552 | - | 530-547 | CBS domain |
| Lsa040900.1 | BLR09 (2) | 576 | - | - | Pentatricopeptide repeat domain |

^1^ according to the Lettuce Genome Resource <http://lgr.genomecenter.ucdavis.edu/>

^2^ predicted using SignalP 4.1

^3^ predicted using TMHMM Server v.2.0

^4^ predicted using InterProScan
