# Supplementary material for: Multiple downy mildew effectors target the stress‐related NAC transcription factor LsNAC069 in lettuce
Source: Plant J. 2019 Jul 4;99(6):1098–115. doi: 10.1111/tpj.14383 (PMC9545932; doi:10.1111/tpj.14383)
Supplement: Supplementary file 18 — Table S3. Primers. [file TPJ-99-1098-s014.docx]

**Supplemental Table 3, Primers used in this study**

| **Primers with BP cloning sites for prey gene amplification**^1^ | | |
| --- | --- | --- |
| *Gene* | *Orientation* | *Sequence* |
| *LsNAC069* | Fwd | *GGGGACAAGTTTGTACAAAAAAGCAGGCTTC*ATGGGTTCTGACTTGATTGAAG |
| *LsNAC069* | Rev | *GGGGACCACTTTGTACAAGAAAGCTGGGTT*TTACCATATACACCTACCCAAT |
| *LsNAC069* pos.523 | Fwd | *GGGGACAAGTTTGTACAAAAAAGCAGGCTTC*ATGAGTGGGCCCGGTCCCAAA |
| *LsNAC069* pos.631 | Fwd | *GGGGACAAGTTTGTACAAAAAAGCAGGCTTC*ATGCGGAATCTGATTCCAATTCC |
| *LsNAC069* pos.802 | Fwd | *GGGGACAAGTTTGTACAAAAAAGCAGGCTTC*ATGGAAAGTTGTGGCGATTTGAAA |
| *LsNAC069* pos.988 | Fwd | *GGGGACAAGTTTGTACAAAAAAGCAGGCTTC*ATGGGTAATGATGAATTTCAAATAAGT |
| *LsNAC069* pos.1188 | Rev | *GGGGACCACTTTGTACAAGAAAGCTGGGTT*TTAACCCGTTCCATTGCCATTAT |
| *LsNAC069* pos.1401 | Rev | *GGGGACCACTTTGTACAAGAAAGCTGGGTT*TTAACCCCTACCCCTACTACTC |

| **Primers with BP cloning sites for hairpinRNA construct amplification**^1^ | | |
| --- | --- | --- |
| *Gene* | *Orientation* | *Sequence* |
| *LsNAC069* | Fwd | *GGGGACAAGTTTGTACAAAAAAGCAGGCTTC*GTGCTTTCCACCTGGTTTC |
| *LsNAC069* | Rev | *GGGGACCACTTTGTACAAGAAAGCTGGGTT*GCCCGACCTTGGTAATAAAC |
| *LsNAC069* | Fwd | *GGGGACAAGTTTGTACAAAAAAGCAGGCTTC*GAGGAGGAATGGAGTGATGA |
| *LsNAC069* | Rev | *GGGGACCACTTTGTACAAGAAAGCTGGGTT*AATTCCGCCAAAGACCCGA |

^1^Sequences in italic represent attB cloning sites

| **Primers for sequencing of hairpinRNA constructs in the destination vector** | | |
| --- | --- | --- |
| *Name* | *Orientation* | *Sequence* |
| pHellsgate12 promoter | Fwd | GGGATGACGCACAATCC |
| pHellsgate12 terminator | Rev | GAGCTACACATGCTCAGG |
| pHellsgate12 intron | Rev | CCGAATTCCTCGAGACCAC |
| pHellsgate12 intron | Fwd | AGGGTCCTAACCAAGAAAATG |

| **Primers for qRT-PCR** |  |  |
| --- | --- | --- |
| *Gene* | *Orientation* | *Sequence* |
| *LsNAC069* | Fwd | ATAATGGCAATGGAACGGG |
| *LsNAC069* | Rev | AGAAGCTGGAGTTGTTGGT |
| *LsNAC091* | Fwd | CATCCAGTCAACAGTGCAAA |
| *LsNAC091* | Rev | GGCACCTCCTCCACACAA |
| Lettuce *ACTIN* (Lsat_1_v5_gn_8_116260.1) | Fwd | CTATCCAGGCTGTGCTTTCC |
| Lettuce *ACTIN* (Lsat_1_v5_gn_8_116260.1) | Rev | ACCCTTCGTAGATCGGGACT |
| *BLN04* | Fwd | AGGGGACATGCTGTATATGG |
| *BLN04* | Rev | TGCTCTCGACATGGTGGTT |
| *BLR05* | Fwd | AGCAAGAACGAGAAAAAGGAAA |
| *BLR05* | Rev | ACAGGCAAAACGGAAGACA |
| *BLR08* | Fwd | CCACACCCTATCCAACTCTC |
| *BLR08* | Rev | ACGGTAATTCGTGCTTCG |
| *BLR09* | Fwd | TGAATTGGAAGAGCGAGGAGGA |
| *BLR09* | Rev | GATGCCGTAGCAAGCAGAGA |
| *B. lactucae ACTIN* | Fwd | GCGAGAAATTGTGCGTGATA |
| *B. lactucae ACTIN* | Rev | ACTCGGCTGCAGTCTTCATT |
