# Supplementary material for: Multiple downy mildew effectors target the stress‐related NAC transcription factor LsNAC069 in lettuce
Source: Plant J. 2019 Jul 4;99(6):1098–115. doi: 10.1111/tpj.14383 (PMC9545932; doi:10.1111/tpj.14383)
Supplement: Supplementary file 19 [file TPJ-99-1098-s015.docx]

**Supporting Information Legends**

**Figure S1. Alignment of yeast isolated prey inserts to LsNAC069.** Graphical representation of the LsNAC069 coding sequence in grey with the position of the NAC and transmembrane domain. Prey inserts were amplified from yeast clones and Sanger sequenced with a forward primer. Reads were aligned to LsNAC069 as indicated with black lines below the LsNAC069 coding sequence. Prey inserts were expected to extend to the end of the LsNAC069 sequence as is indicated with grey dotted lines. **Figure S2. Expression of LsNAC069 and effectors BLR05, BLR08, BLR09 and BLN04 during *B. lactucae* infection.**

(a) Transcript abundance of *B. lactucae* effectors BLN04, BLR05, BLR08, BLR09 and BLN04 and (b) transcript abundance of *LsNAC069* in mock and *B. lactucae* inoculated samples. and (c) *B. lactucae* actin. *L. sativa* cv. Olof seedlings were harvested 3 hours, 1 day, 3 days and 6 days after infection with race Bl:24. LsNAC069 and *B. lactucae* ACTIN expression levels were calculated as ΔCt values relative to *L. sativa* ACTIN. Effector expression levels were calculated as ΔCt values relative to *B. lactucae* ACTIN. Data of a single experiment with three biological replicates is depicted (mean + SE). Data are representative for three independent experiments.

**Figure S3. Subcellular localization of *B. lactucae* effector BLR08 in *N. benthamiana***. CFP-BLR08 was co-expressed with RFP-tagged ER lumenal marker and forms ring-like structures of various sizes. Scale bars indicate 10 µm.

**Figure S4. B. lactucae effectors BLR05, BLR09 and CFP-LsNAC069 predominantly label the ER membrane in lettuce.** YFP-BLR05, RFP-BLR09 and CFP-LsNAC069 were expressed in L. sativa cv. Olof. The two effectors localize to the ER membrane in lettuce. CFP-LsNAC069 cells were treated with MG132 for 12 h before observation. Scale bars indicate 10 µm.

**Figure S5. Phylogenetic relationship of NAC proteins in lettuce.** Multiple alignments were generated using Clustal Omega. A Neighbor-Joining tree was constructed in MEGA 7.0 using pairwise gap deletion with 1000 bootstrap replicates. Classification into subfamilies is indicated. Asteriks (*) indicate NAC proteins with a single C-terminal transmembrane domain.

**Figure S6. Graphical representation of potato and Arabidopsis NACs used in Y2H.** The putative single C-terminal TMD (purple) and N-terminal NAM domain (blue) are indicated.

**Figure S7. Western blotting of CFP-LsNAC069 and truncations.** Increased stability in Western blotting was observed for the CFP-LsNAC069 truncations in comparison to the full CFP-LsNAC069. Without MG132 treatment the full CFP-LsNAC069 is not visible after the Western blot detection. Asterisk indicates full-length proteins; empty arrow indicates free CFP. Rubisco was used as loading reference.

Table below the Western blots shows the theoretical protein size (with and without CFP) and the observed protein size of the different truncations expressed in planta. N.d. = no experiments performed.

**Figure S8. MG132 has a stabilizing effect on the full LsNAC069.** CFP-tagged LsNAC069, LsNAC069^ΔNAC^ and CFP-LsNAC069^267-497^ localize at the ER. Over expression of the full LsNAC069 without MG132 treatment induces ER stress and deformation of nuclei. MG132 has a stabilizing effect on the full LsNAC069, increases visibility of the ER and reduces ER body formation. In contrast, no effect of MG132 has been observed during confocal microscopy on the truncated LsNAC069^ΔNAC^, LsNAC069^267-497^ and LsNAC069^174-467^. In contrast to LsNAC069^ΔNAC^ and LsNAC069^267-497^, the LsNAC069^174-467^ without the transmembrane domain localizes nuclear-cytosolic. Scale bar indicates 10 µm.

**Figure S9. LsNAC069 and effectors BLR05 and BLR09 localize to the ER.** Fluorescent fusions of LsNAC069 and effector proteins were transiently expressed in *N.benthamiana*. Z-stacks were imaged with the Zeiss 700 and used for 3D projection (image J). Scale bars indicate 10 µm.

**Figure S10. Co-expression of LsNAC069^ΔNAC^ with *B. lactucae* effectors and ER marker in *N. benthamiana.*** CFP-LsNAC069^ΔNAC^ co-localizes with YFP-BLR05, YFP-BLR09 and the RFP-ER marker. YFP-BLR05 also localize distinguishable in the Golgi. Scale bars indicate 10 µm. Images were taken with the Laica SP8, 100x objective, oil emersion.

**Figure S11. V8 control does not induce relocalization of CFP-LsNAC069^ΔNAC^ to the nucleus.** All samples were treated with proteasome inhibitor MG132. Confocal settings were identical between samples. Shown is the fluorescence intensity inside the nucleus. Bars represent the mean SD from n≥13 images per treatment. Statistical differences were assessed using one-way ANOVA with *post-hoc* Tukey testing. The V8 control was indicated with an asterisk (*).

**Figure S12. Expression of proteins during translocation experiments.** Western blotting was used to confirm expression of CFP-LsNAC069^ΔNAC^, CFP-LsNAC^174-467^ and HA-tagged effectors in the relocalization experiments. Additionally, to the co-expression of BLR05 and BLR09, also BLR08 was included in the Western blot to confirm stable expression and size difference of the effectors in planta.

Empty arrow, free CFP; monomer of LsNAC069^ΔNAC^ was indicated with an asterisk. The effector bands were indicated with coloured arrows, corresponding to the colour of the effector names indicated in the treatment scheme below the Western blots.

**Figure S13. *LsNAC091* transcript levels.** Transcript levels of the possible off-target *LsNAC091* in untransformed Wendell plants (control) and T2 lines harbouring hpRNA construct 1 and 2. Transcript levels are relative to lettuce actin (Lsat_1_v5_gn_8_116260.1). Each boxplot contains the values of 4-6 plants.

**Figure S14. Nuclear accumulation of LsNAC069^ΔNAC^ induced by PEG is inhibited in the presence of *B. lactucae* effectors.** (a) Localization of CFP-LsNAC069^ΔNAC^ and CFP-LsNAC069^174-467^ in *N. benthamiana* leaf sections. PEG (5% v/v) treatment induces relocalization of CFP-LsNAC069^ΔNAC^. (b) Quantification of arithmetic mean fluorescence in the nucleus. Co-expression with single effectors HA-BLR05 and HA-BLR09 significantly inhibits PEG induced relocalization, but the strongest effect was observed with co-expression of both effectors. The PEG treated LsNAC069^ΔNAC^ expressing samples group together with LsNAC069^174-467^. Effector co-expression significantly reduces relocalization to the nucleus.

All samples were treated with proteasome inhibitor MG132. Confocal settings were identical between samples. The scale bar indicates 10 µm. Bars represent the mean SD from n≥13 images per treatment. Statistical differences were assessed using one-way ANOVA with *post-hoc* Tukey testing.

**Figure S15. *LsNAC069* silencing reduces wilting effects during drought stress at 48 h but plants adjust to the wild type phenotype at 72 h.** Representative pictures of drought stress effect on RNAi lines (T3) at 0 h, 48 h and 72 h, with n≥6 biological replicates. Scale bar indicates 10 cm. Though *LsNAC069* silenced plants show less wilting at 48 h, the phenotype at 72 h of water deprivation is comparable to the untransformed control.

**Table S1. Targets identified in Y2H screening.**

**Table S2. Overview of lettuce NAM domain containing genes.**

**Table S3. Primers.**
